# Diagnosis of Bovine Digital Dermatitis: Exploring the Usefulness of Indirect ELISA

**DOI:** 10.3389/fvets.2021.728691

**Published:** 2021-11-01

**Authors:** João Sucena Afonso, Georgios Oikonomou, Stuart Carter, Helen E. Clough, Bethany E. Griffiths, Jonathan Rushton

**Affiliations:** ^1^Department of Livestock and One Health, Institute of Infection, Veterinary & Ecological Sciences, University of Liverpool, Liverpool, United Kingdom; ^2^Department of Infection Biology & Microbiomes, Institute of Infection, Veterinary & Ecological Sciences, University of Liverpool, Liverpool, United Kingdom

**Keywords:** digital dermatitis, dairy cattle, aetiology, ELISA, diagnosis, United Kingdom

## Abstract

The precision by which animal diseases are diagnosed affects our ability to make informed decisions with regards to animal health management, from a clinical and economic perspective. Lameness is a major health condition in dairy cattle. The underlying causes of lameness include bovine digital dermatitis (BDD), which is reported as one of the main causes of infectious lameness in dairy cattle. Presently, the gold standard for BDD diagnosis in dairy cattle is visual inspection of lifted hooves—a labour intensive and subjective method. Research has suggested that *Treponema* spp. are the main pathogens associated with the establishment of BDD. We explored the potential of indirect enzyme-linked immunosorbent assay (ELISA) as a diagnostic serological tool in the identification of cows at different stages of BDD. Additionally, we evaluated the predictive power of this diagnostic tool on the future occurrence of BDD lesions. A total of 232 cows from three farms were used in the study. Serum samples and hoof health data were collected at three time points: ~ 30 days pre-calving, around calving, and approximately 30 days post-calving. The mean absorbance from the ELISA test was compared across different clinical presentations of BDD as assessed by visual inspection of the hooves according to the M-stage classification system. A transition model was developed to estimate the probability of lesion occurrence in time *t* + *1* based on the spectrophotometer (absorbance) reading in time *t*. The mean absorbance reading for both IgG1 and IgG2 anti-*Treponema* antibodies was associated with disease presence—apart from M4.1 lesions, animals with no lesions had a lower mean when compared to animals with lesions regardless of the score. Additionally, the mean absorbance reading of animals with active lesions was higher when compared to animals with no lesions. However, the anti-*Treponema* antibody assays failed to identify disease presence in a consistent manner. Moreover, indirect ELISA readings were not a predictor of the future occurrence of BDD lesions. In conclusion, although the levels anti-*Treponema* antibodies were associated with disease presence, the ELISA test failed to detect disease unequivocally and had no predictive value in the future occurrence of BDD lesions.

## Introduction

Lameness is the second most important health condition in dairy cattle in the UK in terms of production losses and the most important welfare issue (1). Lameness is a symptom rather than a disease which can have different aetiologies, from infectious to non-infectious causes (2).

Bovine digital dermatitis (BDD) is a leading cause of infectious lameness in British dairy cattle, causing ulcerative skin lesions, with significant impact on animal production and welfare. The impact of BDD is associated with the stage of the disease. Acute ulcerative phases are more likely to result in a change in the behaviour of the cow due to pain, leading to a reduction in milk yield and fertility. Animals chronically affected by digital dermatitis can perpetuate the disease in the herd, acting as pathogen reservoirs and contributing to the establishment of an endemic nature of the ailment, which can result in premature culling of animals and added costs to control and eradicate the disease (3, 4). Identifying the stage of the disease is thus important to inform the treatment and control strategy (5) and to allow for the proper estimation of the economic impact of BDD.

Different methods are currently used to capture data related to BDD. Herd mobility scoring is a screening tool widely used to identify animals afflicted by lameness, followed by clinical investigation of the underlying cause and treatment. Yet mobility scoring is subjective and prone to intra- and inter-observer bias (2, 6). Additionally, the presence of BDD lesion(s) is not always associated with lameness, which could lead to the underreporting of the problem (7). The reference for detecting and classifying BDD is the clinical observation of lifted hooves in a foot trimming chute. In addition to being labour-intensive and expensive, this unavoidable routine, meant for adequate cleaning and examination to be carried, causes stress to the animal (8, 9). Additionally, some cases with less obvious lesions can be overlooked (10). As such, researchers have suggested alternative approaches for monitoring BDD, including visual inspection during milking routine at the parlour (8, 9), and investigated the use of infrared thermography to identify animals with lesions (11). Unfortunately, these tend to have a lower diagnostic capacity when compared to the reference (12).

Research has suggested that *Treponema* species are a key pathogen triggering the pathogenic cascade that leads to the establishment of the disease (13–15). The immunologic response to the presence of these pathogens can be assessed through serology which could stand as a more objective and practical alternative tool of identifying animals with BDD when compared to the current reference diagnostic method (16). A study by Frössling et al., (17) found that the presence of serum anti-*Treponema* antibodies assessed through indirect ELISA could be used with high sensitivity and specificity for the identification of BDD presence at animal and herd levels. Additionally, the results from serology with milk samples from the bulk tank showed good agreement with those from individual cows, suggesting a potential use as a screening tool at herd level (17). The usefulness of indirect enzyme-linked immunosorbent assay (ELISA) as a diagnostic tool in identifying BDD clinical stages has been previously assessed in dairy heifers through the measurement of anti-*Treponema* antibodies. The study found that the mean anti-*Treponema* antibody titres for animals experiencing a DD lesion for the first time increased by 56% when compared to results before the onset of the disease. Additionally, animals treated with oxytetracycline for a DD acute lesion had their anti-*Treponema* antibodies titres decreased to levels closer to those of animals without DD lesions after an average of 223 days (18).

The main aim of this paper was to assess whether indirect ELISA is suitable for the diagnosis and severity assessment of bovine digital dermatitis. It also explored the value of the test as a predicting tool of the future occurrence of BDD lesion by hypothesizing a time lag between the exposure to *Treponema* spp. and the development of clinical signs of disease. Finally, we made use of the available data to explore risk factors for the occurrence of BDD.

## Materials and Methods

Ethical approval for the study was granted by the University of Liverpool Research Ethics Committee (reference VREC466). ASPA regulated procedures were conducted under a Home Office Project License (Reference Number: PPL 70/8330).

### Farm Characteristics and Animals

A cohort of dairy cattle from three commercial dairy farms in the North West of England and North Wales were followed from September 2016 to August 2017. Farms were conveniently recruited for their proximity to the research institute and for their willingness to take part in the study. The farms' characteristics are described in more detail in a previous study (19).

On farm 1, animals were housed in concrete cubicles with different mattress types and bedded with sawdust. Automatic scrapers removed manure from pen passageways two or three times per hour. The milking parlour and part of the collecting yard had rubber floor matting. Dry cows were housed in grooved concrete floors with deep straw bedding. Youngstock spent summer on pasture and winter housed in cubicle sheds.

Cows were housed in cubicles on farm 2. High yielding cows were kept in sheds with concrete cubicles with mats and shallow sand beds. For low yielding and freshly calved cows, cubicles had deep sand bedding. As with farm 1, automatic scrapers removed manure from pen passageways two or three times per hour. The milking parlour and the collecting yard were concreted with no floor matting. Dry cows were kept on pasture during summer months and housed in deep sanded cubicles in winter. Youngstock were housed in concrete cubicles with straw.

On farm 3, animals were housed in deep sand bedded cubicles. Pen passageways were scraped three times a day with a tractor. The milking parlour and main race had floor matting. Dry cows and youngstock were kept separately, each in its own unit. Dry cows were housed in deep sand bedded cubicles.

All farms had foot trimming routines. In farms 1 and 3, all animals were foot trimmed at drying off and around 60 days in milk (DIM); whereas in farm 2, cows were only trimmed at drying off. Footbathing was used in all farms. In farm 1, animals were footbathed once a week with 3% formalin and twice a week with 4% copper sulphate as they exited the milking parlour. Farms 2 and 3 only used 3% formalin in their footbaths. In farm 2, cows were footbathed three times per week, and in farm 3, once a day.

### Blood Samples

A blood sample was obtained from the coccygeal vein at three time points-−30 days pre-calving, within 7 days from calving and 30 days post-calving. Blood samples were collected into a plain 5 ml vacutainer (BD Vacutainer Serum Tube, BD, USA) and allowed to clot at room temperature before being placed on ice. Samples were transferred to the laboratory within a few hours from collection where clotted blood was subsequently centrifuged (Sigma 3–16kl, Sigma Laborzentrifugen, Germany) at 2,500 rpm at 20°C for 10 min. Aliquots of serum were then frozen at −20°C.

### Clinical Data Collection

Health data were recorded on the day blood samples were collected by one of the two trained observers (GO and BG), and mobility score (MS) and body condition score (BCS) were recorded. The methods for collecting data on the mentioned parameters are detailed by Griffiths et al. (19). Cows were mobility scored when walking on a flat surface using the four-point AHDB scale [0 = good mobility, 3 = severely impaired mobility] (20). The BCS was assessed through the Penn State Method [1 = very thin, 5 = obese] (21). For the clinical observation of foot lesions, animals were restrained in a trimming chute and their hoofs were lifted for assessment. Digital dermatitis lesions were classified according to their stage using a six-point scale (Table 1).

**Table 1:** Classification of digital dermatitis lesions according to macroscopic aspect.

**Stage**	**Clinical descriptor**
M1	Small (<2 cm across) focal active state. Circumscribed lesion. Surface is moist, ragged, mottled red–grey with scattered small (~1 mm diameter) red foci
M2	Larger (>2 cm across) ulcerative active stage. Extensively mottled red–grey. Can be painful upon manipulation
M3	Healing stage. Typically seen within a few days after antibiotic treatment. The ulcerated surface is now transformed to a dry brown, firm rubbery scab. No pain on manipulation
M4	Chronic stage. Surface is raised by tan, brown, black, rubbery, irregular, proliferative hyperkeratotic growths that vary from papilliform to mass-like projections
M4.1	Chronic stage with small active painful M1 focus
M5	No sign of pre-existing lesion. Normal skin

*Adapted from (22); the terms acute ulcerative were changed to ulcerative active*.

Additionally, the presence of other hoof health events was assessed in all four hooves during the foot trimming performed at 60 days in milk and classified according to the ICAR Claw Health Atlas (23) as per below:

- Bovine digital dermatitis
- Interdigital dermatitis
- Interdigital hyperplasia
- Interdigital phlegmon
- Sole haemorrhage/bruising
- Sole ulcer
- White line disease
- Other lesions

### Laboratory Procedures

#### Preparation of Treponema Antigens

*Treponema* antigens were prepared as per Dhawi et al. (24) by sonication of protein concentrate obtained from a culture of *Treponema* Group 2 bacteria, previously isolated (*Treponema phagedenis* T320A) from digital dermatitis lesions (25).

#### Indirect ELISA

Indirect ELISA tests were conducted on serum samples to assess the presence of anti-*Treponema* antibodies (subclasses IgG1 and IgG2). The protocol is outlined below but can be found in more detail in the Supplementary Material alongside with the list of reagents used.

Non-activated 96-well microtitre ELISA plates were coated with sonicate protein extracts from *T. phagedenis* T320A resuspended in PBS, pH 7.2; 5 μg protein/ml. The plates were incubated for 1 h at 37°C and overnight at 4°C inside a lidded humid sandwich box. The liquid in the wells was discarded and the plates thoroughly washed three times with PBS-Tween 20 (0.05%) to remove unbounded antigen. Sera dilutes of cows were prepared on the same day of the coating of the plates, at a dilution of 1/1,000, by adding 10 μl of serum sample to 990 μl of PBS-Tween 20 (0.05%) in an Eppendorf, and kept in the fridge overnight at 4°C.

After stirring the Eppendorf in the vortex stirrer, cow serum samples (100 ul, 1:100 dilution in PBS-Tween 20) were pipetted into the wells in duplicate. Diagram of the ELISA plate can be found in the protocol in the Supplementary Material. Blank control wells got 100 μl of PBS-Tween 20 (0.05%). Positive and negative control serum samples were identified from a first set of experiments and used throughout for comparison with test sera to ensure data consistency.

The plates were incubated with sera samples for 1 h at 37°C and plates were washed with PBS-Tween 20 (0.05%). One hundred μl of mouse anti-bovine IgG (Bio-Rad; diluted 1:10,000) were added to each well. The plates were again incubated at 37°C for 1 h.

The plates were washed as before, and 100 μl of peroxidase conjugated goat anti-mouse IgG (Sigma), at a dilution of 1:10,000, was added to each well. The plates were incubated for 1 h at 37°C.

After washing the plate, 100 μl of 3,3′,5,5′-tetramethylbenzidine (Interchim) was added to each well for detecting the antibody–conjugate reaction. The plates were left for 20 min in the dark at room temperature for 20 min or until the blue colour was saturated enough to prevent the loss of linearity between the colour saturation and antibody titre. After this period 100 μl of hydrochloric acid at 0.5 M (Sigma) were added to each well, and the results were read in a multi-well ELISA plate reader (Multiskan, Titertek) at 405 nm. Each plate was read three times and the average taken. The absorbance readings obtained from serum samples of animals known to be negative for BDD signs (BDD-free farms) were used for validating plates. These sample derived from another piece of research (26). Plates were considered valid if the average from the negative control duplicates were below the cut-off defined as the mean of the results from the animal known to be negative plus three standard deviations. The variation between duplicates was calculated. All samples for which duplicates had a variation over 20% were considered invalid, and samples were retested. Data was normalized taking the positive control of each plate as reference (27).

### Data Management and Parameters

Microsoft Excel (2016) was used to collate the data and create the data set (28). The health data collected with regards to BDD was used to create new binary variables to explore their association with the readings from the indirect ELISA as follows, with each BDD lesion record assumed to be a case:

- Presence of BDD lesion(s) (yes/no)
- Presence of active DD lesions regardless of the number (yes/no)—the definition of an active lesion followed the lesion classification system and descriptors by Berry et al. (22), thus M1, M2, and M4.1 lesions were classified as active
- Presence of active lesions considering the number (no lesions/one lesion/more than one lesion)

Additionally, BCS was used to create a three-point scale variable (29). The three-point scale BCS variable assumed the following intervals—below 2.5, between 2.5 and 3.5 and above 3.5. Moreover, animals were grouped according to their lactation into three categories: first lactation, second lactation, third lactation, or above. A binary variable for the ELISA test was also created based on the absorbance readings for IgG1 and IgG2. The cut-off value was defined by mean of the results from the animal known to be negative plus three standard deviations.

Furthermore, animals were classified across five categories according to lesion recording during the study period as per below:

**Table:** 

**BDD health category**	**Data collection time point**		
	**Pre-calving**	**Around calving**	**Post-calving**
Healthy	No lesion	No lesion	No lesion
Deteriorating	No lesion	No lesion	Lesion(s)
	No lesion	Lesion(s)	Lesion(s)
Recovering	Lesion(s)	No lesion	No lesion
	No lesion	Lesion(s)	No lesion
	Lesion(s)	Lesion(s)	No lesion
Ever ill	Lesion(s)	Lesion(s)	Lesion(s)
Other	Lesion(s)	No lesion	Lesion(s)

Lastly, a categorical variable was created based on the disease progression which considered three categories—no change, improved, and worsen—by comparing lesion score in time *t* with that at time *t - 1*. A shift from no lesion in time *t - 1* to any other score represented a worsening condition. An active lesion with no signs of chronicity in time *t - 1* could have an improvement or a worsening of the health condition. If the animal had an M1 lesion in time *t - 1*, its health condition could improve (no lesion or M3 in time *t*) or deteriorate (M2, M4, and M4.1 in time *t*). Similarly, if the animal had an M2 lesion in time *t - 1*, it could improve (no lesion or M3 in time *t*) or get worse (M4 and M4.1 in time *t*). If an M4 lesion had been detected in *t - 1*, the disease could progress (M4.1 in time *t*) or recede (no lesion or M3 in time *t*). In case of a chronic lesion with signs of acuteness (M4.1) lesion in *t - 1*, the health condition could only improve (no lesion, M3 or M4 in time *t*). If the cow had the same lesions score in consequent time periods, no change was assumed. The correspondence of values across the different combinations can be found in Supplementary Table 2.

### Statistical Analysis

The analyses were conducted using RStudio statistical software [version 1.3.1073] (30, 31).

#### Risk Factors for BDD Occurrence

A logistic regression model was developed using the package *finalfit* (32). The explanatory variables—farm, data collection time period, assessor, ELISA result for IgG1 result as a binary variable, ELISA result for IgG2 as a binary variable, mobility score, lameness, three-point scale body condition score, parity—were individually assessed for their relation with the outcome of interest—BDD presence. Those with a *p*-value equal or lower than 0.1 were considered for the multivariable logistic regression model. Animal identification number was used to deal with within-subject repeated measurements as a random effect. A backward elimination approach followed—explanatory variables were eliminated one at the time, starting with the one with the highest value, and the impact in the model's performance appreciated through the Akaike's Information Criteria (AIC), with lower values being rewarded. If the model had a lower AIC, the variable was eliminated from the model (33).

#### Association Between Absorbance and BDD Lesion Score

The distribution of the absorbance readings for IgG1 and IgG2 was assessed for normality using the Shapiro–Wilk normality test. Boxplots on the mean absorbance for IgG1 and IgG2 readings across the different BDD lesion scores were produced using the package *ggplot2* (34). The statistical significance of the observed differences was assessed by the Kruskal–Wallis test when more than two categories existed. If the *p*-value for the Kruskal–Wallis test was lower than 0.05, the Dunn's test for multiple comparisons, with the “Holm” method to adjust for the *p*-value, was conducted to assess the statistical significance of the observed differences between the different groups (35). The paired samples Wilcoxon test was used when only two categories existed.

#### Predictive Power of Indirect ELISA for Future Occurrence of BDD Lesion

Acknowledging the usefulness of anticipating the future occurrence of bovine digital dermatitis based on the current readings of the indirect ELISA test, we assessed the correlation between the ELISA test result in time *t - 1* and the presence of BDD lesion(s) in time *t*.

We fit two models, the first relating the transition probability of each animal at time *t* to (a) the farm to which it belongs and (b) positivity or not to antibody titre IgG1 at the previous measurement at time *t - 1*; and the second relating the transition probability of each animal at time *t* to (a) the farm to which it belongs and (b) positivity to an antibody titre IgG2 at the previous measurement at time *t - 1*. The response *Yi* is a binary variable representing whether or not animal *i* has a lesion at time *t*, conditional on them having no lesion and a positive ELISA test at time *t - 1*, and takes the value 1 if the animal has a lesion at time *t* and 0 if not. Times under consideration are *t* = 1 (30 days pre-calving), *t* = 2 (around calving), and *t* = 3 (30 days post-calving). The model is fitted within the generalised linear modelling framework thus:


$$\begin{array}{l} Y_{i}\sim \;Bernoulli\left(P_{01i}\right) \end{array}$$

with


$$\begin{array}{l} logit\left(P_{01i}\right)=\left(\frac{P_{01i}}{1-P_{01i}}\right)=at+b_{1}x_{i1t}+b_{2}x_{i2} \end{array}$$

where *t* again denotes visit (1, 2, or 3), *xi1t* the positivity of animal *i* to the relevant antibody (IgG1 or IgG2) at visit (*t - 1*), and *xi2* indicating the farm to which animal *i* belongs (one, two, or three, included here as a fixed effect since these are the only farms of interest to this study).

We also assessed whether the percentage variation in the absorbance reading from *t* to *t - 1* was associated with disease evolution by comparing the mean percentage variation across the different disease progression groups.

## Results

### Data Exclusion

Out of the 773 available samples, only 696 were used in the analysis, which resulted in 48 animals being excluded from the analysis. Farm 3 had the most animals excluded from the analysis−17 out of 48 (35%), whereas farm 2 was the one with the least number of animals removed from the analysis−12 out of 48 (25%). Farm 1 had 13 (27%) animals excluded from the analysis. Six animals (13%) were excluded as a result of untraceable samples (Supplementary Table 3).

Samples were excluded for different reasons, and details can be found in Supplementary Tables 4, 5. Missing samples were the main cause for samples to be excluded from the analysis accounting for 83% of the 77 dropped samples.

### Descriptive Statistics

In total, 696 samples were processed corresponding to 232 animals, the majority of which (101) belonged to farm 1 (43.5%). The least represented dairy unit was farm 2 with 50 animals (21.6%). Farm 2 had more cows in their third or more lactation when compared to the farm 1 and 3 (56.0% against 44.6 and 35.8%, respectively). Farm 2 had the highest proportion of cows in the “deteriorating” (28%) and “ever ill” (16%) categories compared to the other farms. More than half of the cows in farms 1 and 3 were classified as “healthy” according to BDD progression (59.4 and 51.8%, respectively), whereas only 36% of the cows in farm 2 were in the same category (Table 2).

**Table 2:** Distribution of sample population according to lactation number and BDD progression type per farm.

**Variable**	**Category**	**Farm 1**		**Farm 2**		**Farm 3**	
		**%**	***N***	**%**	***N***	**%**	***N***
**Lactation number**							
	1	32.7	33	24.0	12	27.2	22
	2	22.8	23	20.0	10	37.0	30
	3 or more	44.6	45	56.0	28	35.8	29
**BDD progression**							
	Healthy	59.4	60	36.0	18	51.8	42
	Deteriorating	6.9	7	28.0	14	16.0	13
	Recovering	15.8	16	16.0	8	18.5	15
	Ever ill	11.8	12	16.0	8	12.3	10
	Other	5.9	6	4.0	2	1.2	1

Most of the data collection was conducted by one investigator (BG) regardless of the farms and time point. Farm 1 had a lower prevalence of lameness when compared to farms 2 and 3 across all the time points. The most significant difference was at pre-calving when 10.5% of the cows were lame in farm 1, whereas 29.2 and 27.8% had the same problem in farm 2 and 3, respectively. Looking at body score condition (BSC) in a three-point scale, the majority of cows (78% or more) were classified between 2.5 and 3.5 across all farm and time points. Farm 2 had 22% of cows with a BSC over 3.5 at pre-calving, roughly 10% more when compared to farm 1 and 3 (Table 3).

**Table 3:** Summary statistics of categorical variables per data collection time point across farms.

**Variable**	**Category**	**Farm 1**						**Farm 2**						**Farm 3**					
		Pre C*		AC*		Post C*		**Pre C**		**AC**		**Post C**		**Pre C**		**AC**		**Post C**	
		**%**	***N***	**%**	***N***	**%**	***N***	**%**	***N***	**%**	***N***	**%**	***N***	**%**	***N***	**%**	***N***	**%**	***N***
**Assessor**																			
	BG	100.0	101	69.3	70	87.1	88	92.0	46	76.0	38	76.0	38	72.8	59	79.0	64	64.2	52
	GO	0.0	-	30.7	31	12.9	13	8.0	4	24.0	12	24.0	12	27.2	22	21.0	17	35.8	29
**3-Point scale BCS**																			
	<2.5	3.0	3	8.9	9	21.8	22	0.0	-	4.0	2	18.0	9	2.5	2	2.5	2	13.6	11
	Between 2.5 and 3.5	84.2	85	87.1	88	78.2	79	78.0	39	92.0	46	82.0	41	85.2	69	88.9	72	86.4	70
	>3.5	12.9	13	4.0	4	0.0	-	22.0	11	4.0	2	0.0	-	12.3	10	8.6	7	0.0	-
**Mobility score**																			
	0	55.4	56	22.8	23	23.8	24	26.0	13	6.0	3	6.0	3	9.9	8	14.8	12	11.1	9
	1	28.7	29	48.5	49	54.5	55	42.0	21	50.0	25	52.0	26	60.5	49	51.9	42	58.0	47
	2	9.9	10	27.7	28	20.8	21	26.0	13	30.0	15	32.0	16	27.2	22	32.1	26	28.4	23
	3	0.0	-	0.0	-	0.0	-	2.0	1	2.0	1	0.0	-	0.0	-	1.2	1	0.0	-
	No data	5.9	6	1.0	1	1.0	1	4.0	2	12.0	6	10.0	5	2.5	2	0.0	-	2.5	2
**Lameness**																			
	Normal	89.5	85	72.0	72	79.0	79	70.8	34	63.6	28	64.4	29	72.2	57	66.7	54	70.9	56
	Lame	10.5	10	28.0	28	21.0	21	29.2	14	36.4	16	35.6	16	27.8	22	33.3	27	29.1	23
**BDD lesion**																			
	No	70.3	71	77.2	78	75.2	76	68.0	34	58.0	29	52.0	26	74.1	60	75.3	61	70.4	57
	Yes	29.7	30	22.8	23	24.8	25	32.0	16	42.0	21	48.0	24	25.9	21	24.7	20	29.6	24
**BDD lesion score**																			
	M1	26.7	8	26.1	6	4.0	1	37.5	6	33.3	7	16.7	4	25.0	5	45.0	9	20.8	5
	M2	3.3	1	0.0	-	0.0	-	37.5	6	14.3	3	8.3	2	25.0	5	10.0	2	12.5	3
	M3	63.3	19	56.5	13	80.0	20	12.5	2	52.4	11	54.2	13	20.0	4	20.0	4	29.2	7
	M4	6.7	2	13.0	3	16.0	4	6.3	1	0.0	-	16.7	4	20.0	4	15.0	3	29.2	7
	M4.1	0.0	-	4.3	1	0.0	-	6.3	1	0.0	-	4.2	1	10.0	2	10.0	2	8.3	2

*Pre C, Pre-Calving; AC, Around calving; Post C, Post-Calving.

Considering the observation of clinical lesions throughout the study, bovine digital dermatitis was more prevalent in farm 2, particularly around and post-calving−42.0% and 48.0% compared to 22.8 and 24.8% in farm 1 and 24.7 and 29.6% in farm 3, respectively. Disease profile was different across the different farms. Farm 1 had most of their cows with BDD in the healing stage (M3) throughout the study period (63.3, 56.5, and 80.0% at pre-calving, around calving, and post-calving, respectively). Farms 2 and 3 had 75% and 50% of their affected animals with active lesions at pre-calving. As the production cycle progressed, farm 2 revealed an increase in M3 stage animals (52.4 and 54.2% for around and post-calving, respectively) and a reduction of animals with M1 and M2 lesions (47.6 and 25.0% for around and post calving, respectively). Farm 3 had the highest proportion of animals with active lesions at around calving (55%) and post-calving (33.3%) when compared to the other farms. It also had the highest proportion of animals affected with BDD with signs of chronicity (M4 and M4.1 lesions) across all farms throughout the study period−30.0, 25.0, and 37.5% at pre-, around, and post-calving, respectively (Table 3).

Farm 2 and 3 had considerably higher records of claw horn disruptive lesions at 60 days in milk (CHDL) when compared with farm 1. Roughly half of the animals had at least a lesion in farm 2 and 3 (48.0% and 50.6%, respectively); whereas in farm 1, a CHDL was recorded 22.8% of the times (Table 4).

**Table 4:** Distribution of claw horn disruptive lesions collected at 60 days in milk across the three farms.

**Variable**	**Category**	**Farm 1**		**Farm 2**		**Farm 3**	
		**%**	***N***	**%**	***N***	**%**	***N***
**SU** ^***a***^							
	No	91.1	92	78.0	39	92.6	75
	Yes	8.9	9	22.0	11	7.4	6
**SH** ^***b***^							
	No	90.1	91	80.0	40	74.1	60
	Yes	9.9	10	20.0	10	25.9	21
**WLD** ^***c***^							
	No	92.1	93	76.0	38	67.9	55
	Yes	7.9	8	24.0	12	32.1	26
**CHDL** ^***d***^							
	No	77.2	78	52.0	26	49.4	40
	Yes	22.8	23	48.0	24	50.6	41

*^a^Sole Ulcer.*

*^b^Sole Haemorrhage.*

*^c^White Line Disease.*

*^d^Claw Horn Disruption Lesion*.

### Risk Factors for Bovine Digital Dermatitis (Inferential Statistics)

In the univariate analysis farm, positiveness in the ELISA test for both IgG1 and IgG2 antibodies, mobility score, lameness, and parity were identified as risk factors (*p* ≤ 0.1) for the presence of DD lesions, regardless of the affected hoof, and accepted for the multivariate model. Animals in farm 2 had twice as much the risk of having BDD when compared with farm 1 (OR 1.94, 95% CI 1.28–2.94), whereas cows from farm 3 were at the same risk of having the disease as farm 1 (OR 1.01, 95% CI 0.69–1.49). Animals testing positive in the ELISA for IgG1 or IgG2 antibodies were at higher risk of having DD lesions when compared with those testing negative−4.06 the odds (95% CI 2.88–5.74) and 2.50 (95% CI 1.70–3.67) the odds, respectively. Lame animals had 1.49 times the odds (95% CI 1.03–2.15) of having DD lesions when compared with non-lame animals. Additionally, animals with mobility score (MS) one or two were at higher risk of having BDD when compared to animals with MS = 0 (1.51 with a 95% CI 0.97–2.40, and 2.06 OR with a 95% CI 1.26–3.42, respectively). Animals in their second parity were at lower risk of having BDD compared to animals in their first parity (0.66 OR, 95% CI 0.42–1.02; Table 5).

**Table 5:** Risk factors for the presence of DD lesions regardless of the affected hoof in a univariate logistic regression model.

**Variable**	**Categories**	**Bovine digital dermatitis**				**Unadjusted odds ratio (95% CI, *p*-value)**
		**No Lesions**		**DD Lesions**		
		**%**	***N***	**%**	***N***	
**Farm**						
	1	73.9	224	26.1	79	1 (reference)
	2	59.3	89	40.7	61	**1.94 (1.28–2.94**, ***p*** **=** 0.002)*
	3	73.7	179	26.3	64	1.01 (0.69–1.49, *p* = 0.944)
**Data collection time point**						
	Pre	71.1	165	28.9	67	1 (reference)
	Fresh	72.4	168	27.6	64	0.94 (0.63–1.41, *p* = 0.757)
	1 month	68.5	159	31.5	73	1.13 (0.76–1.68, *p* = 0.544)
**Assessor**						
	BG	70.1	390	29.9	166	1 (reference)
	GO	72.9	102	27.1	38	0.88 (0.57–1.32, *p* = 0.559)
**IgG1 binary**						
	Negative	81.5	358	18.5	81	1 (reference)
	Positive	52.1	134	47.9	123	**4.06 (2.88–5.74**, ***p*** **<** 0.001)*
**IgG2 binary**						
	Negative	74.8	416	25.2	140	1 (reference)
	Positive	54.3	76	45.7	64	**2.50 (1.70–3.67**, ***p*** **<** 0.001)*
**Body condition score−3 categories**						
	<2.5	72.9	35	27.1	13	1 (reference)
	Between 2.5 and 3.5	70.5	415	29.5	174	1.10 (0.58–2.08, *p* = 0.770)
	>3.5	70.2	33	29.8	14	0.96 (0.64–1.40, *p* = 0.823)
**Mobility score**						
	0	78.8	119	21.2	32	1 (reference)
	1	71.1	244	28.9	99	**1.51 (0.97–2.40**, ***p*** **=** 0.076)*
	2	64.4	112	35.6	62	**2.06 (1.26–3.42**, ***p*** **=** 0.005)*
	3	66.7	2	33.3	1	1.86 (0.08–20.01, *p* = 0.617)
**Lameness**						
	No	73.5	363	26.5	131	1 (reference)
	Yes	64.4	114	35.6	63	**1.53 (1.06–2.21**, ***p*** **=** 0.016)*
**Parity group**						
	1	67.2	135	32.8	66	1 (reference)
	2	75.7	143	24.3	46	**0.66 (0.42–1.02**, ***p*** **=** 0.065)*
	3+	69.9	214	30.1	92	0.88 (0.60–1.29, *p* = 0.510)

*Statistically significant when p <0.05.

When running the multivariable logistic regression, the model with parity group and ELISA result for IgG1 performed the best when considering the AIC. Animals testing positive for IgG1 antibodies had approximately four times the odds of having a DD lesion when compared to animals testing negative (4.29 OR, 95% CI 2.97–6.28). Animals in their third or more lactation had roughly half the odds of having a DD lesion when compared with cows in their first lactation (0.56 OR, 95% CI 0.36–0.86; Table 6).

**Table 6:** Odds ratio for the presence of DD lesions regardless of the affected hoof in a multivariable logistic regression model.

**Variable**	**Categories**	**Adjusted odds ratio (95% CI)**	***p*-value**
**IgG1 binary**			
	Negative	1 (reference)	-
	Positive	4.34 (2.99–6.36)	<0.001*
**Parity group**			
	1	1 (reference)	-
	2	0.65 (0.40–1.06)	0.085
	3+	0.56 (0.36–0.86)	0.008*

*Statistically significant when p < 0.05.

### Correlation Between ELISA Results and BDD Lesions

**Figure 1:**
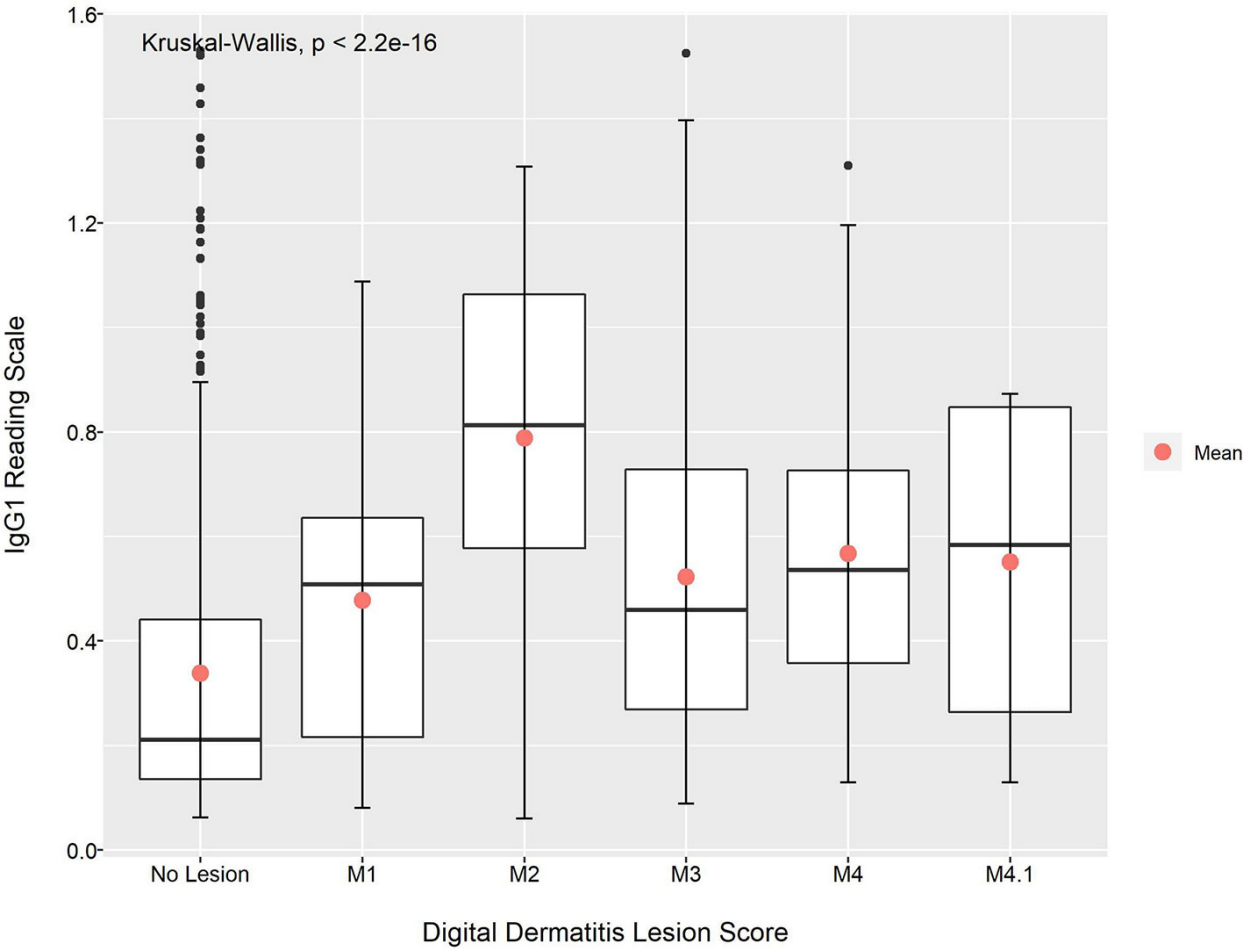
Mean absorbance reading for IgG1 anti-*Treponema* antibodies across the different DD lesion scores.

**Figure 2:**
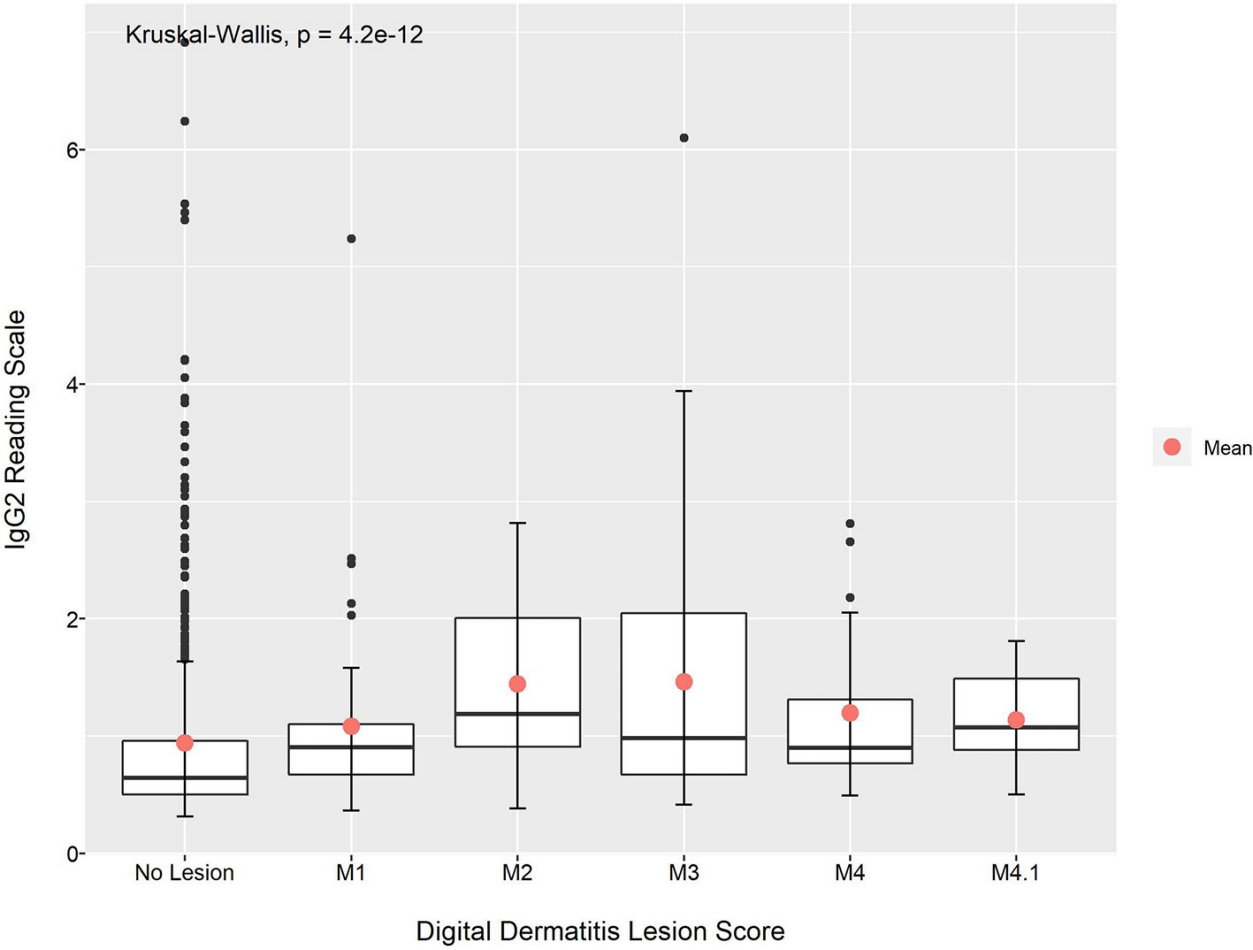
Mean absorbance reading for IgG2 anti-*Treponema* antibodies across the different DD lesion scores.

The Shapiro–Wilk normality test indicated that the IgG1 and IgG2 absorbance readings were not normally distributed. The Kruskal–Wallis test indicated that there were statistically significant differences in the mean absorbance for IgG1 and IgG2 across the BDD lesions scores (*p* < 0.01 for both IgG1 and IgG2; Figures 1, 2). This was also observable across the different data collection time points (Supplementary Figures 1, 2).

With the exception of animals with M4.1 lesions, the Dunn's test pairwise comparison revealed that the mean absorbance reading for IgG1 and IgG2 anti-*Treponema* antibodies in animals with no lesion was lower when compared to animals with BDD lesions (*p* < 0.05; Table 7). Looking at the data per time period, the animals with no lesions had a lower mean absorbance reading for IgG1 and IgG2 when compared to animals with M3 lesions at around calving and post-calving (*p* < 0.05). At pre-calving, the mean absorbance reading for both IgG1 and IgG2 was found to be significantly different between animals with no lesions and animals with M2 lesions (*p* < 0.05; Table 7).

**Table 7:** Pairwise comparison of mean absorbance readings across different BDD lesion scores.

**Variable**	**Nonparametric pairwise comparison (Bonferroni-adjusted** ***p*****-value)**														
	**NL^**1**^ vs. M1**	**NL vs. M2**	**NL vs. M3**	**NL vs. M4**	**NL vs. M4.1**	**M1 vs. M2**	**M1 vs. M3**	**M1 vs. M4**	**M1 vs. M4.1**	**M2 vs. M3**	**M2 vs. M4**	**M2 vs. M4.1**	**M3 vs. M4**	**M3 vs. M4.1**	**M4 vs. M4.1**
IgG1	0.000*	0.000*	0.000*	0.000*	0.155	0.082	1.000	1.000	1.000	0.138	1.000	1.000	1.000	0.790	1.000
IgG1-t1^a^	0.1064	0.000*	0.674	0.146	1.000	0.570	1.000	1.000	0.919	0.075	1.000	1.000	1.000	1.000	1.000
IgG1-t2^b^	0.107	0.368	0.000*	0.473	1.000	1.000	1.000	1.000	0.961	1.000	1.000	1.000	1.000	1.000	1.000
IgG1-t3^c^	0.481	0.059	0.001*	0.017*	0.323	1.000	1.000	1.000	1.000	1.000	1.000	0.998	1.000	1.000	1.000
IgG2	0.005*	0.000*	0.000*	0.005*	0.226	1.000	1.000	1.000	1.000	1.000	1.000	1.000	0.982	1.000	1.000
IgG2-t1	0.246	0.001*	1.000	0.110	1.000	0.707	1.000	1.000	1.000	0.051	1.000	1.000	0.647	1.000	0.859
IgG2-t2	0.506	1.000	0.000*	1.000	1.000	0.969	0.554	1.000	1.000	1.000	1.000	1.000	1.000	1.000	1.000
IgG2-t3	1.000	1.000	0.000*	0.247	1.000	1.000	1.000	1.000	0.976	1.000	1.000	1.000	1.000	1.000	1.000

*^1^No lesion.*

*^a^Pre-calving.*

*^b^Around calving.*

*^c^Post-calving.*

^*^Significant p-values (<0.05) in bold.

**Figure 3:**
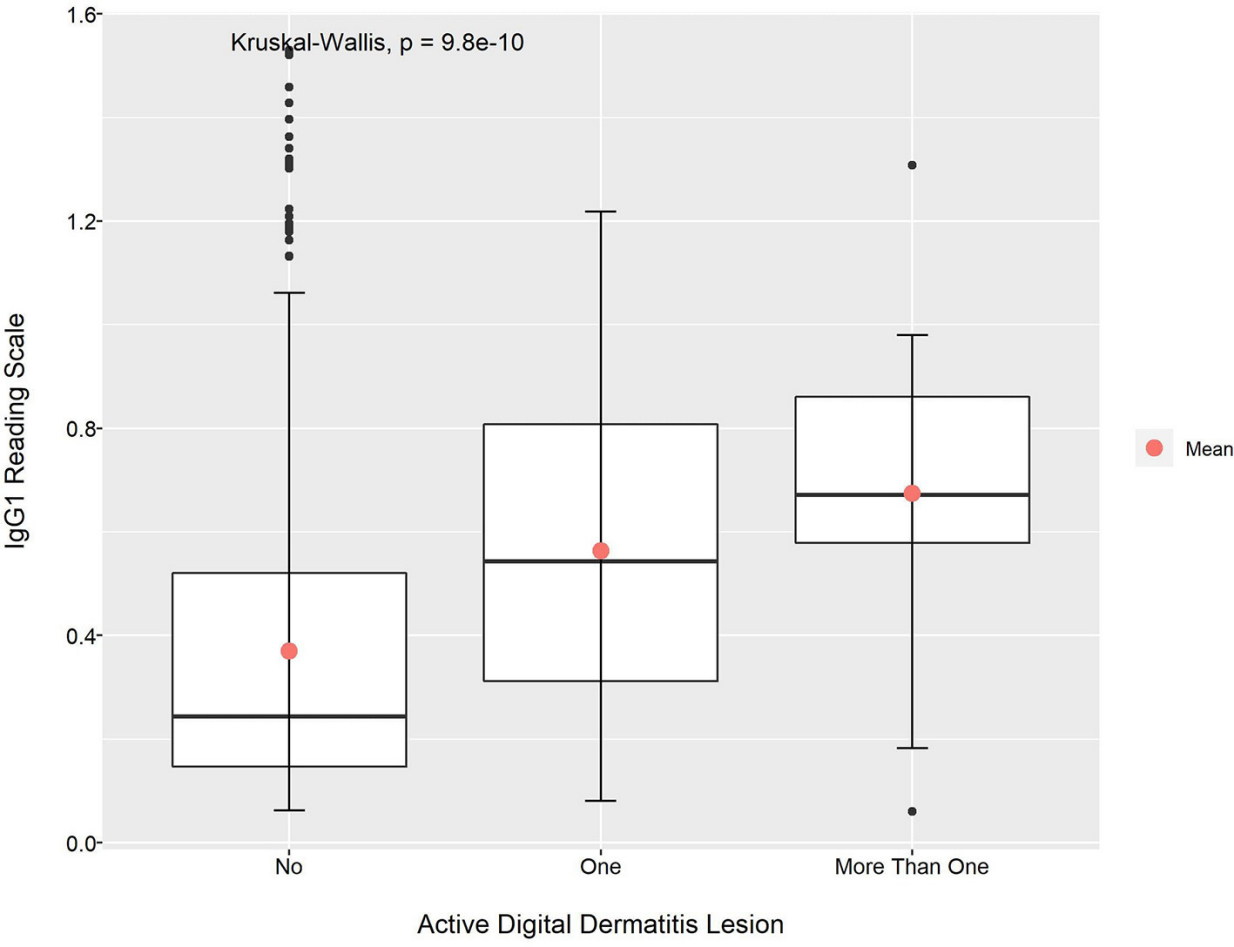
Mean absorbance reading for IgG1 anti-*Treponema* antibodies according to the presence of active DD lesion.

**Figure 4:**
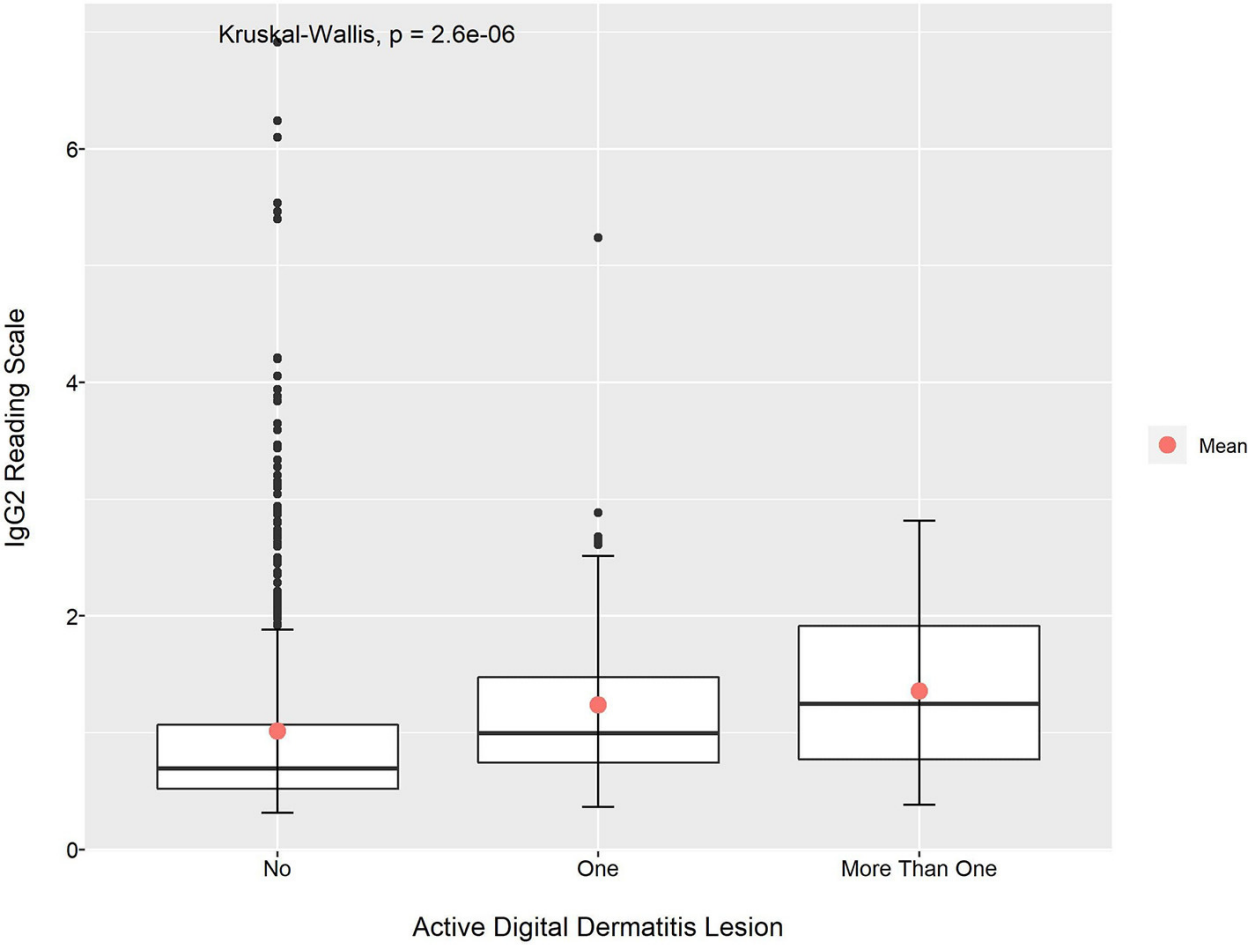
Mean absorbance reading for IgG2 anti-*Treponema* antibodies according to the presence of active DD lesion.

Looking at the relationship between mean absorbance readings for IgG1 and IgG2 anti-*Treponema* antibodies and the presence of an active BDD lesion(s), the Kruskal–Wallis test also showed that the observed differences were significant (*p* < 0.01 for both cases; Figures 3, 4). Apart from the mean absorbance reading for IgG2 at post-calving, the pairwise comparison using Dunn's test indicated that the mean absorbance reading for IgG1 and IgG2 in animals with no lesions was significantly lower than animals with one active lesions (*p* < 0.05). When comparing the mean absorbance reading for IgG1 and IgG2 between the group of animals with no active lesions and the group of animals with more than one active lesions, the later had a higher value considering all data point (*p* < 0.05). This was also observable for IgG1 at pre- and post-calving and for IgG2 at pre-calving (*p* < 0.05; Table 8 and Supplementary Figures 3, 4).

**Table 8:** Pairwise comparison of mean absorbance readings across different categories regarding number of active BDD lesions.

**Variable**	**Nonparametric pairwise comparison**		
	**(Bonferroni-adjusted** ***p*****-value)**		
	**NL^**1**^ vs. one active lesion**	**NL vs. two or more active lesions**	**One active lesion vs. two or more active lesions**
IgG1	0.000*	0.000*	0.413
IgG1-t1^a^	0.000*	0.013*	0.607
IgG1-t2^b^	0.001*	0.607	0.622
IgG1-t3^c^	0.028*	0.023*	0.218
IgG2	0.000*	0.036*	0.931
IgG2-t1	0.000*	0.006*	0.349
IgG2-t2	0.005*	0.846	0.292
IgG2-t3	0.397	0.696	0.899

*^1^No lesion.*

*^a^Pre-calving.*

*^b^Around calving.*

*^c^Post-calving.*

^*^Significant p-values (<0.05) in bold.

### Correlation Between Anti-*Treponema* Antibodies and BDD Health Category

When looking at the mean absorbance reading for IgG1 and IgG2 anti-*Treponema* antibodies across the different BDD health categories, the Kruskal–Wallis test indicated significant differences between groups when considering all the data and when looking across the different data collection time points (*p* < 0.05; Supplementary Figures 5–8).

The Dunn's test for pairwise comparison revealed that the group of animals labelled as *healthy* had lower mean absorbance reading for both IgG1 and IgG2 when compared to the group of animals classified as *deteriorating* and *ever ill*, regardless of whether all data was considered, or when looking at the data per time period (*p* < 0.05). This was also evident when comparing the group *recovering* with the *ever ill* group—apart from the absorbance reading for IgG1 at pre calving, the later had higher mean absorbance readings for both IgG1 and IgG2 (*p* < 0.05). The *ever ill* group had higher mean absorbance reading for IgG1 and IgG2 reading when compared to any of the other health categories (*p* < 0.05; Table 9).

**Table 9:** Pairwise comparison of mean absorbance readings across different BDD health categories.

**Variable**	**Nonparametric pairwise comparison (Bonferroni-adjusted** ***p*****-value)**									
	**Healthy vs. Recov^**1**^**	**Healthy vs. Deterior^**2**^**	**Healthy vs. Ever ill**	**Healthy vs. Other**	**Recov vs. Deterior**	**Recov vs. Ever ill**	**Recov vs. Other**	**Deterior vs. Ever ill**	**Deterior vs. Other**	**Ever ill vs. Other**
IgG1	0.001*	0.000*	0.000*	0.106	0.093	0.000*	0.911	0.014*	0.380	0.007*
IgG1-1^a^	0.187	0.005*	0.000*	0.653	0.802	0.052	1.000	0.546	0.759	0.699
IgG1-t2^b^	0.117	0.006*	0.000*	1.000	1.000	0.009*	0.793	0.112	0.804	0.118
IgG1-t3^c^	0.274	0.000*	0.000*	0.564	0.341	0.013*	0.993	0.717	0.773	0.317
IgG2	0.100	0.000*	0.000*	0.129	0.020*	0.000*	0.438	0.008*	0.608	0.009*
IgG2-t1	1.000	0.013*	0.000*	0.985	0.406	0.001*	0.721	0.240	0.920	0.262
IgG2-t2	0.512	0.008*	0.000*	1.000	0.580	0.002*	0.964	0.141	0.771	0.132
IgG2-t3	0.941	0.005*	0.000*	0.806	0.489	0.021*	0.915	0.879	0.688	0.685

*^1^Recovering.*

*^2^Deteriorating.*

*^a^Pre-calving.*

*^b^Around calving.*

*^c^Post-calving.*

^*^Significant p-values (<0.05) in bold.

### Predictive Power of ELISA Test in Future Occurrence of BDD Lesion(s)

The results from the generalised linear model-based transition models showed limited predictive power of the ELISA test upon the future occurrence of BDD lesions for both IgG1 and IgG2 anti-*Treponema* antibodies. Farm 2 had higher occurrence of BDD lesions. When looking at the absorbance readings for IgG1, animals from farm 2 were at higher risk of developing BDD lesions around calving compared to farm 1 (4.03 OR, *p* = 0.02). When considering the absorbance readings for IgG2, farm 2 animals were at higher risk of developing lesions both at around calving and 1 month post-calving when compared to farm 1 (4.16 OR, *p* = 0.016 and 3.42 OR, *p* = 0.023, respectively; Table 10).

**Table 10:** Results from the generalised linear models assessing the predictive power of ELISA test in the future occurrence of BDD lesion(s).

**Model**			**OR (95% CI)**	***p*-value**
**IgG1**				
Predicting occurrence of	IgG1 at	Negative	1 (reference)	-
BDD around calving	pre-calving	Positive	0.91 (0.32-2.40)	0.8467
	Farm	Farm 1	1 (reference)	-
		Farm 2	**4.03 (1.25–13.81)**	**0.0207**
		Farm 3	1.67 (0.55–5.36)	0.3690
Predicting occurrence of	IgG1 around	Negative	1 (reference)	-
BDD post-calving	calving	Positive	1.01 (0.34–2.70)	0.9858
	Farm	Farm 1	1 (reference)	-
		Farm 2	2.73 (0.92–8.04)	0.0664
		Farm 3	1.34 (0.53–3.37)	0.5314
**IgG2**				
Predicting occurrence of	IgG2 at	Negative	1 (reference)	-
BDD around calving	pre-calving	Positive	0.73 (0.19–2.33)	0.6242
	Farm	Farm 1	1 (reference)	-
		Farm 2	**4.16 (1.32–13.99)**	**0.0161**
		Farm 3	1.65 (0.54–5.29)	0.3822
Predicting occurrence of	IgG2 around	Negative	1 (reference)	-
BDD post-calving	calving	Positive	0.37 (0.08–1.26)	0.1502
	Farm	Farm 1	1 (reference)	-
		Farm 2	**3.42 (1.17–10.03)**	**0.0231**
		Farm 3	1.37 (0.54–3.47)	0.4987

*Significant p-values (<0.05) in bold*.

The mean variation in the absorbance readings from *t* to *t - 1* for both IgG1 and IgG2 was not associated with disease progression, neither when considering all data, nor when looking at the data per time period (*p*-value for Kruskal–Wallis > 0.05; Supplementary Figures 9–12).

## Discussion

This paper explored the usefulness of measuring anti-*Treponema* antibody titres through indirect ELISA test in the identification of cows at different stages of BDD, and prediction power of this diagnostic tool in the future occurrence of disease.

The results indicated that the absorbance readings from indirect ELISA were significantly associated with BDD profile. With the exception of M4.1 score, animals with no lesions had in general lower mean absorbance readings when compared with the other lesion scores for both IgG1 and IgG2 anti-*Treponema* antibodies. Additionally, the mean absorbance reading in the group of cows with no lesions for both IgG1 and IgG2 anti-*Treponema* antibodies was significantly lower when compared to the group of animals with one active lesion and with two or more active lesions. Furthermore, BDD health category was associated with mean absorbance reading. The groups of animal diagnosed with a BDD lesion in all three time point assessments (e*ver ill*) had significantly higher absorbance mean when compared to any of the other BDD health groups for both IgG1 and IgG2 anti-*Treponema* antibodies. Also the *deteriorating* group had significant higher absorbance mean when compared to the group of animals that had no BDD lesions throughout the study period. Despite the chronic exposure to *Treponema* spp. due to the endemic nature of BDD, these findings suggest that different disease stages lead to different immunologic profiles that can be measured through indirect ELISA allowing for the identification of BDD status in dairy cows. A previous study looked at the immunologic profile across different BDD lesion scores and found that M2 lesions resulted in higher titres compared with other lesions (18). Although no significant differences were observed in the mean readings between the different BDD lesion scores, the *p*-value for some of the pairwise comparisons was close to the significance level (0.05). In a recent study, Holmøy et al. (36) found that ELISA tests could identify anti-*Treponema* antigens in milk samples from the bulk tank. Unfortunately, the diagnostic test did not perform well with regards to sensitivity and specificity, probably due to the disease prevalence (low) and profile (mild symptoms) in the study population (36). Similarly, a weakness of our analysis is that the samples are non-random and originate from three farms only: as such milder cases (lower scores) are more heavily represented than more severe cases (higher scores) in the data set and this may have impacted upon the power of the analysis to detect associations between dependent variables and lesion score. Evaluating the effectiveness of a diagnostic tool such as indirect ELISA in the identification of disease profile at the herd level in a larger (ideally random) sample, which more comprehensively represents the profile of lesion scores across all levels of severity, could result in an objective alternative to the current reference method—visual inspection of lifted hooves.

Farm as a unit was identified as a risk factor for BDD with animals in farm 2 having double the odds of BDD lesions when compared to farm 1. This is reinforced by the findings from the transition model in which farm 2 was a predictor for the future occurrence of BDD lesions. The role of farm management practices has been acknowledged in BDD incidence (37, 38). Foot trimming has also been identified as a measure to reduce BDD incidence (39, 40). Somers et al., reported that animals trimmed at longer intervals (>7 months) were at a higher risk of developing the disease when compared to animals trimmed more frequently (41). As opposed to farms 1 and 3, cows in farm 2 were only trimmed once a year at drying-off. Between farm differences in hygiene levels and in foot bathing practices could also explain these findings. Even though an in-depth investigation would be required to reach valid conclusion as to the observed differences, the distinct farm management practices and hoof health prevention protocol between farms could offer an explanation to the observed dissimilarities.

Literature has indicated that cow-level risk factors impact on BDD occurrence such as parity number, with primiparous cows at higher risk when compared to multiparous (42). This is in line with the results from multivariate model in which cows with three or more lactation were at lower risk of BDD when compared with cows in their first lactation. Different reasons could explain such observation. It may relate to selection of older cows with lesions for culling leading to a reduction of disease prevalence with age (43). Alternatively, the early culling of dairy heifers affected with BDD could promote a selection of animals less susceptible to the disease (5).

Although a correlation between the readings from the indirect ELISA and the different BDD disease presentations was established, the usefulness of the laboratory test in identifying diseased animals as a binary outcome is questionable, as it failed to consistently classify animals with clinical manifestations of disease as positive—out of the 696 test results, 81 were false negatives when testing for the presence of IgG1 and 140 were false negatives when assessing the presence of IgG2. Additionally, the lab test failed to predict in a reliable manner the future occurrence of BDD lesions, limiting its usefulness in the implementation of pre-emptive measures to control disease onset. It must be noted that the endemic nature of BDD means that animals are chronically exposed to *Treponema* spp. and hence antibody levels do not necessarily reflect current infection status (16).

The small sample of farms enrolled in the study might have limited our ability to reach significant results. Given the role of farm management in disease incidence and presentation, it would be interesting to further explore the potential usefulness of indirect ELISA in a larger sample of farms. Additionally, the very low incidence of certain disease stages, particularly the M4.1 stage, might have limited the capacity to find significant differences regarding absorbance readings across the different disease stages. The results that were reached make room for further investigating association between indirect ELISA results and disease stages, and the magnitude of the differences, ideally with a larger dataset.

The implications of disease diagnostic are not just limited to the clinical aspects of animal health management by making sure that adequate and successful treatment and control protocols are adopted. It is also a key aspect regarding the overall picture of disease patterns and associated economic impact—a vital information for the decision-making process regarding the management of animal diseases at farm and higher levels of governance (44, 45). In the absence of such information, it is not possible to determine if allocation of the resources is efficient from an economic or welfare perspective. Having diagnostic methods and tools that, for their subjective nature, underestimate the frequency of disease could impair our ability to properly acknowledge the importance of diseases in the overall scenario of production losses and expenditure related to a given sector of animal production. As such objective methods for identifying animals with impaired productivity and/or welfare with subsequent investigation of the underlying causes are important for informing the clinical management of diseases and the assessment of animal disease impacts.

## Data Availability Statement

The raw data supporting the conclusions of this article will be made available by the authors, without undue reservation.

## Ethics Statement

The animal study was reviewed and approved by the Research Ethics Committee University of Liverpool. Written informed consent was obtained from the owners for the participation of their animals in this study.

## Author Contributions

JA and JR discussed and agreed the approach. JA was responsible for the execution of the lab work, data management and analysis, and writing the manuscript. SC supervised the lab work. Clinical data and serum samples were collected by BG and GO. HC provided assistance for analysing the data. The paper was revised and agreed by the co-authors. All authors contributed to the article and approved the submitted version.

## Conflict of Interest

The authors declare that the research was conducted in the absence of any commercial or financial relationships that could be construed as a potential conflict of interest.

## Publisher's Note

All claims expressed in this article are solely those of the authors and do not necessarily represent those of their affiliated organizations, or those of the publisher, the editors and the reviewers. Any product that may be evaluated in this article, or claim that may be made by its manufacturer, is not guaranteed or endorsed by the publisher.
