# Supplementary material for: Diagnosis of Bovine Digital Dermatitis: Exploring the Usefulness of Indirect ELISA
Source: Front Vet Sci. 2021 Nov 1;8:728691. doi: 10.3389/fvets.2021.728691 (PMC8591176; doi:10.3389/fvets.2021.728691)
Supplement: Supplementary file 1 [file Data_Sheet_1.pdf]

1 Supplementary material

1.1 Indirect ELISA laboratory protocol

1. Preparing reagents
    a. Phosphate Buffered Saline (PBS)
        i. Dilute 10 tablets in 1L of dionised water using a magnetic stirrer
    b. PBS-Tween
        i. Prepared PBS as in point a.
        ii. Add 500µl to the PBS and stir using magnetic pill
    c. Stopping solution - Hydrochloric acid (HCl) 0.5M
        i. Work safely on the Hoote
        ii. Dilute 41.059 ml of HCl to 250 ml of dionised water – add HCl slowly (the reaction is exothermic and will produce heat)
        iii. Add dionised water up to 1L
2. Coating the ELISA plates with AG
    a. Prepare Ag dilutes at a concentration of 5µg per ml of PBS – add 0.54 µl of Ag alicots to 10 µl of PBS
    b. Add 100 µl to each well
    c. Place plate in a lidded sandwich box with a damped paper towel at the bottom as to prevent an excessively dry atmosphere
    d. Place box in incubator at 37° degrees Celsius for one hour
    e. Place box in refrigerator at 4° degrees Celsius for 24 hours
    f. Safely discard content in wells
    g. Wash plates thoroughly
        i. Fill in wells with PBS tween with the dispenser making sure to expel the forming air bubbles out of the wells
        ii. Discard liquid in wells
        iii. Repeat step i. and ii. twice
        iv. After the third washing shake the plate as to remove as much liquid as possible
        v. Place disposable absorbing paper on the bench and tap the plate with well's opening facing the bench on the paper to remove the liquid that remained in the wells
    h. Plates can be used after washing or kept in the refrigerator at 4° degrees Celsius for up to____ before being used
3. Preparing serum dilutes
    a. Stir the Eppendorf for a couple of seconds in the vortex
    b. Add 10 µl of serum sample to 990 µl of PBS tween in an Eppendorf
    c. Stir the Eppendorf
    d. Identify each Eppendorf to match the corresponding serum sample
    e. Keep dilutes in a refrigerator at 4° degrees Celsius and use them within 72 hours of preparation

4. Set the ELISA plate
    a. Serum dilutes
        i. Stir Eppendorf in vortex for a couple of seconds
        ii. Add 100 µl of serum dilute to each well as per diagram below (make duplicates of each sample and control; duplicates go left to right; samples go left to right and top to bottom). Control blanks gets 100 µl of PBS tween.

	1	2	3	4	5	6	7	8	9	10	11	12
A	+	+	S6	S6								
B	-	-	S7	S7								
C	B	B	…	…								
D	S1	S1										
E	S2	S2										
F	S3	S3										
G	S4	S4										
H	S5	S5										

        iii. Place plate in a lidded sandwich box with a damped paper towel at the bottom as to prevent an excessively dry atmosphere
        iv. Place box in incubator at 37° degrees Celsius for one hour
        v. Safely discard content in wells
        vi. Wash plates thoroughly as in line g. of point 2.
    b. Mouse anti-bovine IgG (dilution 1:10000)
        i. Add 1.1 µl of reagent to 11 ml of PBS tween – stir container in vortex
        ii. Add 100 µl to each well. Control blanks get 100 µl of PBS tween.
        iii. Place plate in a lidded sandwich box with a damped paper towel at the bottom as to prevent an excessively dry atmosphere
        iv. Place box in incubator at 37° degrees Celsius for one hour
        v. Safely discard content in wells
        vi. Wash plates thoroughly as in line g. of point 2.
    c. Peroxidase conjugate goat anti-mouse IgG (dilution 1:10000)
        i. Add 1.1 µl of reagent to 11 ml of PBS tween – stir container in vortex
        ii. Add 100 µl to each well. Control blanks get 100 µl of PBS tween.

   iii. Place plate in a lidded sandwich box with a damped paper towel at the bottom as to prevent an excessively dry atmosphere
   iv. Place box in incubator at 37º degrees Celsius for one hour
   v. Safely discard content in wells
   vi. Wash plates thoroughly as in line g. of point 2.

d. Substrate (TMB)
   i. Add 100 µl to each well. Control blanks get 100 µl of PBS tween.
   ii. Place plate in a dark place at room temperature for 20 minutes or until the blue colour is saturated enough – readings over 2.5 are to prevent as the initial linear relation between the colour saturation and the Ab titer begins to plateaux.

e. Stopping solution (HCl)
   i. Add 100 µl to each well

f. Read plate
   i. Turn on ELISA reader and open Ascent software
   ii. Set the measurement mode for continuous
   iii. Set the measurement mode for continuous
   iv. Set the optical density filter for 405 nm (yellow)
   v. Read the plate three times

g. Validating results
   i. Control blank should have a reading near absolute zero
   ii. Duplicates with a variation of over 20% are considered invalid
   iii. Average duplicates

The table below provides a summary of the reagents used.

Table 1. List of reagents used for conducting the indirect ELISA tests

Manufacturer	Reagent	Code	Lot/Batch
**Oxoid**	Phosphate Buffered Saline Tablets (Dulbecco A)	BR 0014 G	-
**Sigma Aldrich**	Tween 20	P1379	SLBV2781
	Anti-Mouse Polyvalent Immunoglobulins (G,A,M)−Peroxidase antibody produced in goat	A 0412	SLBT6258
	Hydrochloric acid 37%	258148	STBJ1296
**Biorad**	Mouse anti Bovine IgG1	MCA 2440GA	1806
	Mouse anti Bovine IgG2	MCA 2441GA	1801
**Interchim**	TMB	UP 664781	T0803P002

1.2 Data management and parameters

Table 2 Combination of lesion score across the different data collection time points and the corresponding value to the categorical variable created for describing disease progression

Lesion score in time *t*	Lesion score in time *t-1*	Value for categorical variable “BDD progression”
No lesion	No lesion	no change
	M1	worsen
	M2	worsen
	M3	worsen
	M4	worsen
	M4.1	worsen
M1	No lesion	improved
	M1	no change
	M2	worsen
	M3	improved
	M4	worsen
	M4.1	worsen
M2	No lesion	improved
	M2	no change
	M3	improved
	M4	worsen
	M4.1	worsen
M3	No lesion	improved
	M1	worsen
	M2	worsen
	M3	no change
	M4	worsen
	M4.1	worsen
M4	No lesion	improved
	M3	improved
	M4	no change
	M4.1	worsen
M4.1	No lesion	improved
	M3	improved
	M4	improved
	M4.1	no change

1.3 Results

1.3.1 Data exclusion

Table 3. Distribution of the animals excluded from the analysis (n=48)

	No of animals excluded due to exclusion of untraceable samples (% of the total)	No of animals excluded due to exclusion of samples without clinical data (% of the total)	No of animals excluded due to missing sample(s) (% of the total)	Total (% of the total)
**Farm 1**	-	1 (2%)	12 (25%)	13 (27%)
**Farm 2**	-	1 (2%)	11 (23%)	12 (25%)
**Farm 3**	-	1 (2%)	16 (33%)	17 (35%)
**No information**	6 (13%)	-	-	6 (13%)

Table 4. Distribution of the samples excluded from the analysis (n=77)

	No of untraceable samples excluded (% of the total)	No of duplicate samples excluded (% of the total)	No of samples excluded due to absent clinical data (% of the total)	No of samples excluded due to missing sample(s) (% of the total)
**Farm 1**	-	2 (3%)	1 (1%)	20 (26%)
**Farm 2**	-	-	2 (3%)	17 (22%)
**Farm 3**	-	-	1 (1%)	27 (35%)
**No information**	7 (9%)	-	-	-
**Total number of excluded samples (% of the total)**	7 (9%)	2 (3%)	4 (5%)	64 (83%)

Table 5. Distribution of the samples excluded from the analysis due to missing sample(s) (n=64)

	No of samples excluded due to missing sample(s) (% of the total)			Total (% of the total)
	30 days pre-calving	Around calving	30 days post-calving	
**Farm 1**	11 (17%)	5 (8%)	4 (6%)	20 (31%)
**Farm 2**	7 (11%)	4 (6%)	6 (9%)	17 (27%)
**Farm 3**	10 (16%)	10 (16%)	7 (11%)	27 (42%)

1.3.2 Correlation between indirect ELISA and other parameters

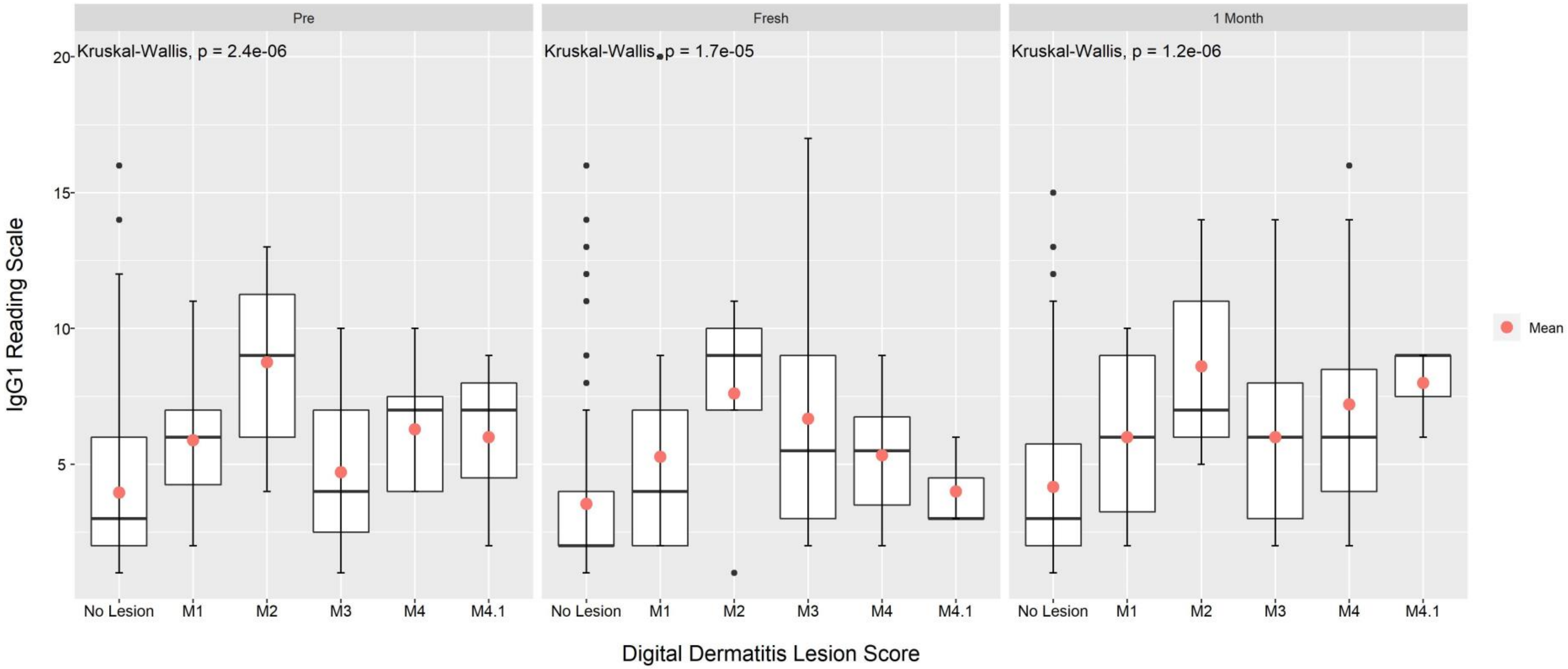

Figure 1. Indirect ELISA IgG1 reading across the different DD lesion scores across the three data-collection time points

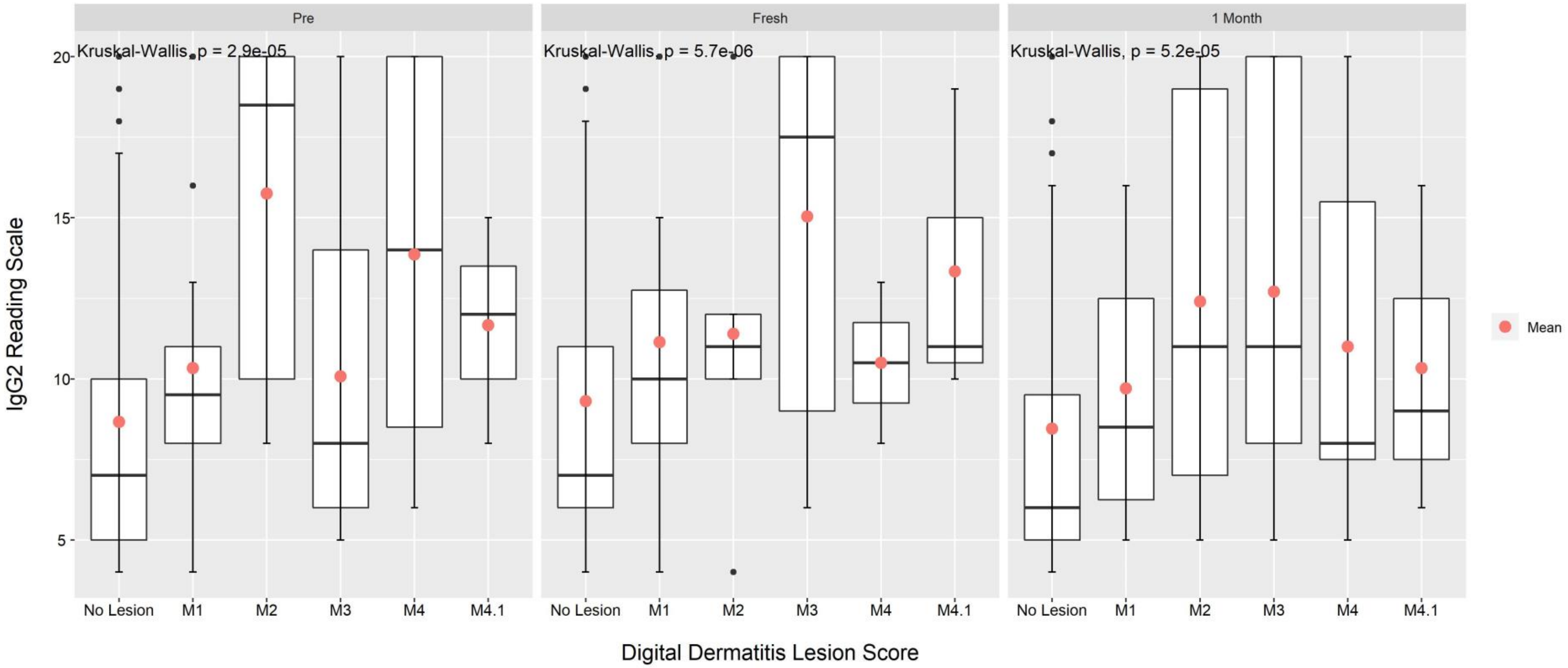

Figure 2. Indirect ELISA IgG2 reading across the different DD lesion scores across the three data-collection time points

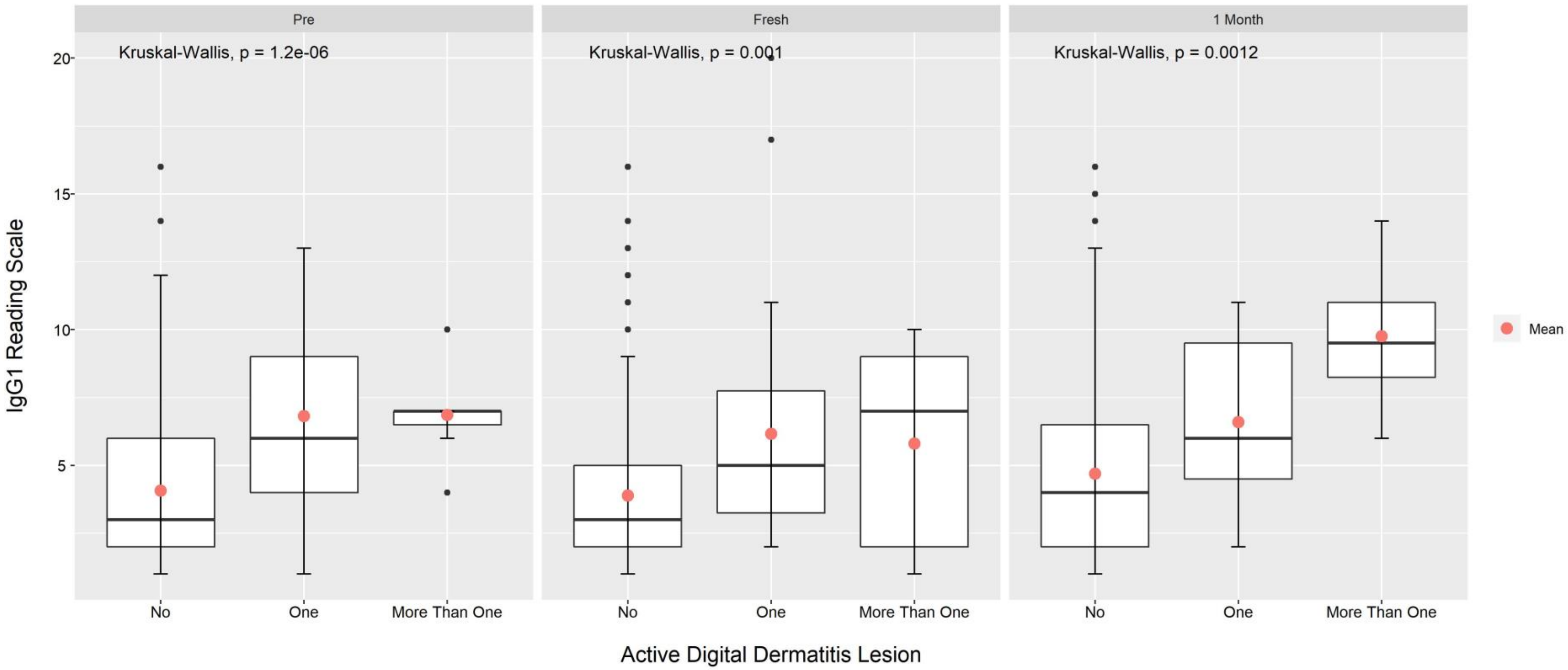

Figure 3. Indirect ELISA IgG1 reading according to presence of active DD lesion across the three data-collection time points

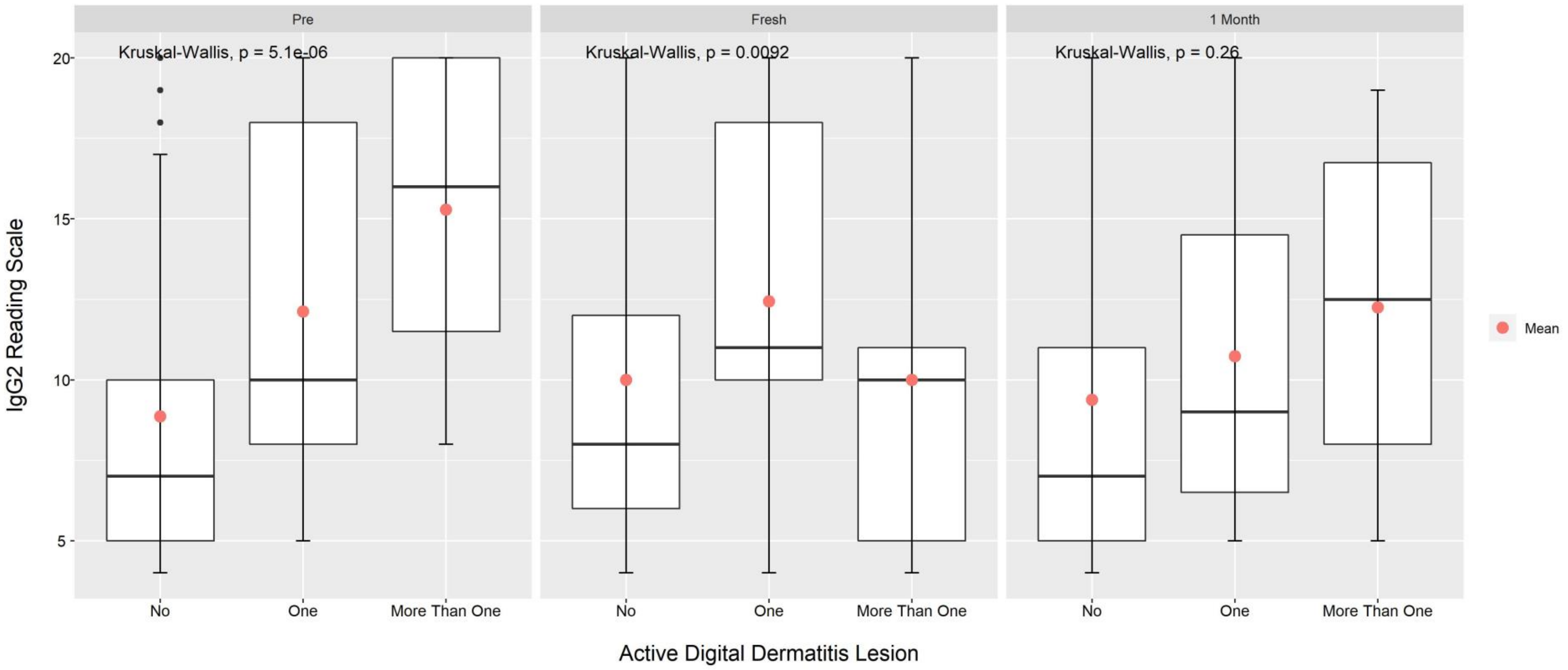

Figure 4. Indirect ELISA IgG2 reading according to presence of active DD lesion across the three data-collection time points

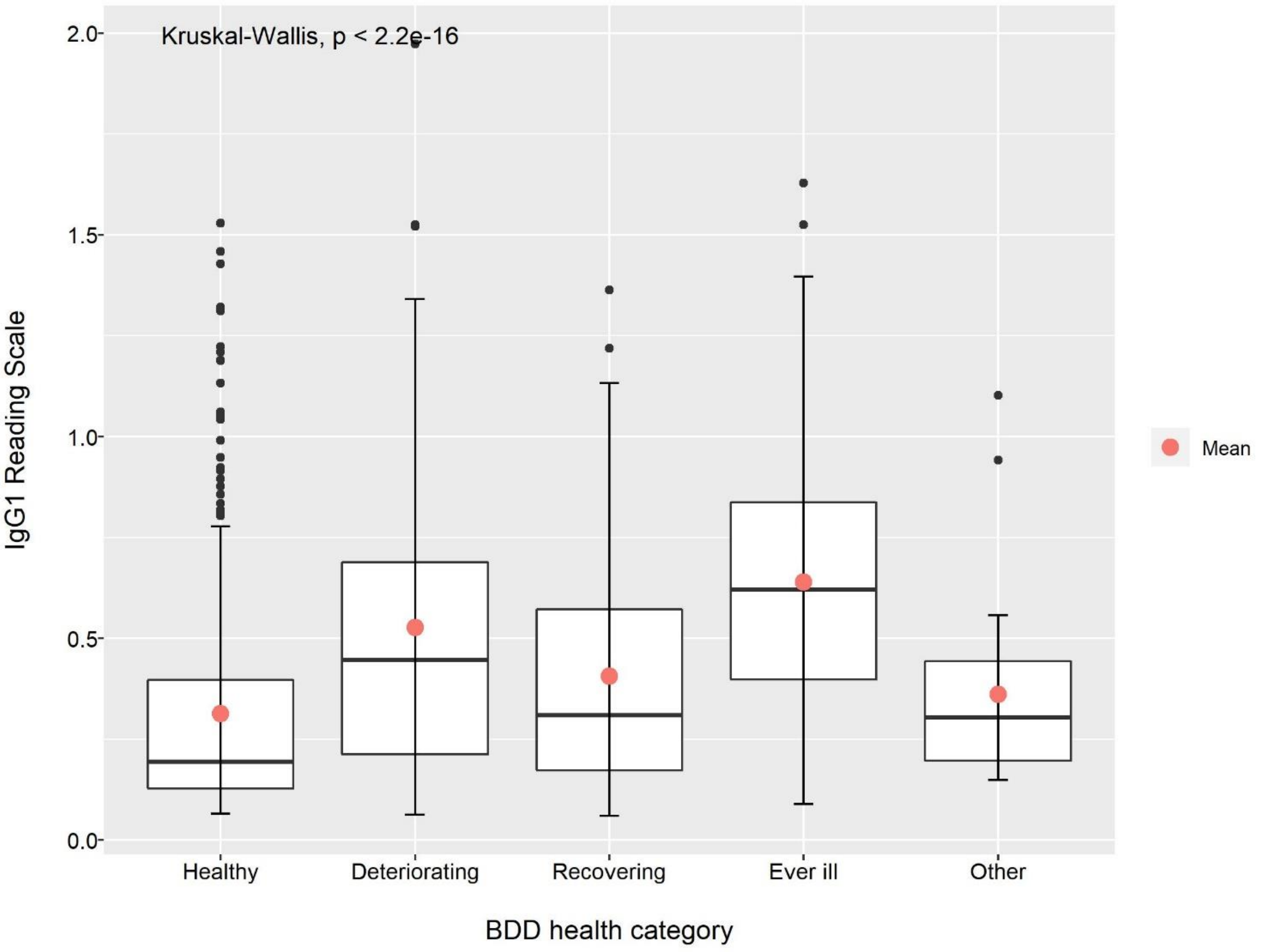

Figure 5. Indirect ELISA IgG1 reading according to BDD health category

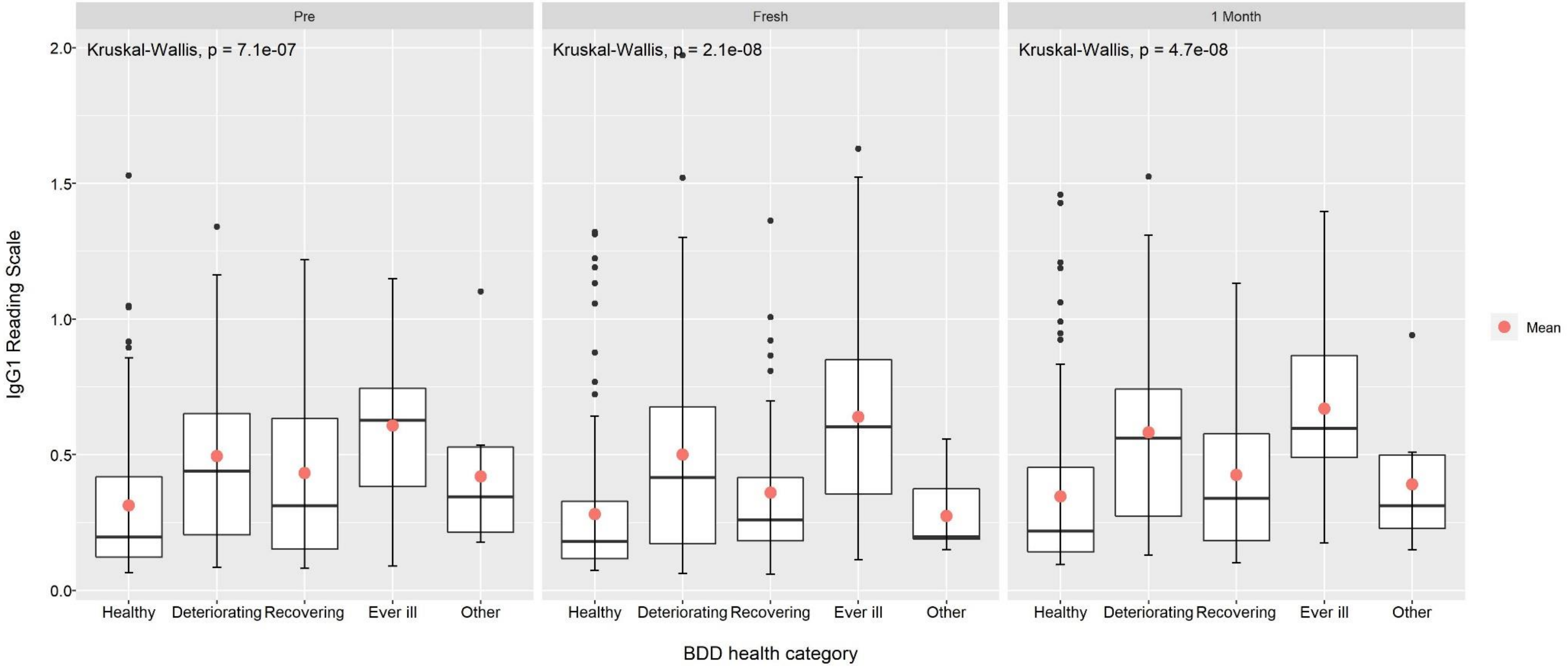

Figure 6. Indirect ELISA IgG1 reading according to BDD health category across the three data-collection time points

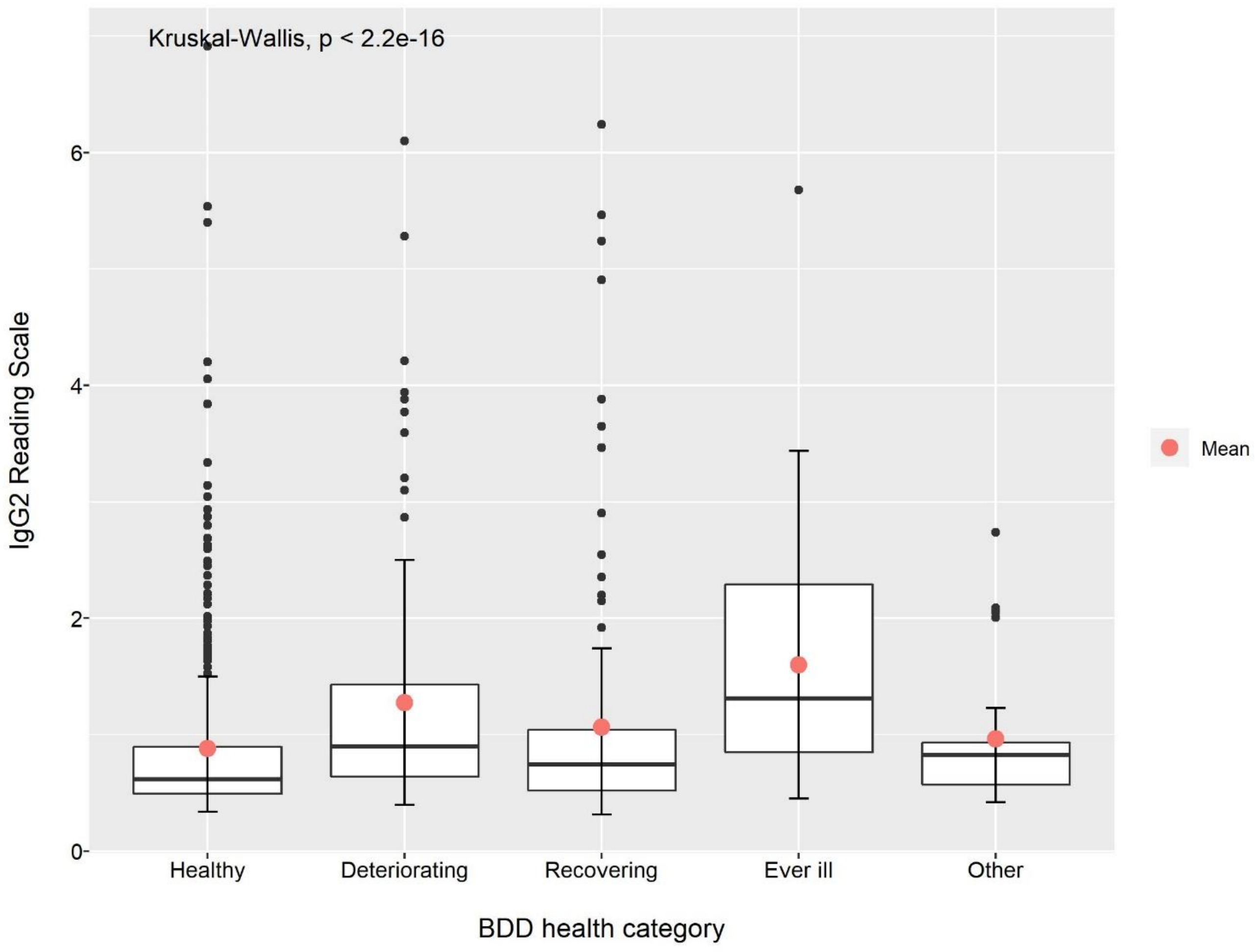

Figure 7. Indirect ELISA IgG2 reading according to BDD health category

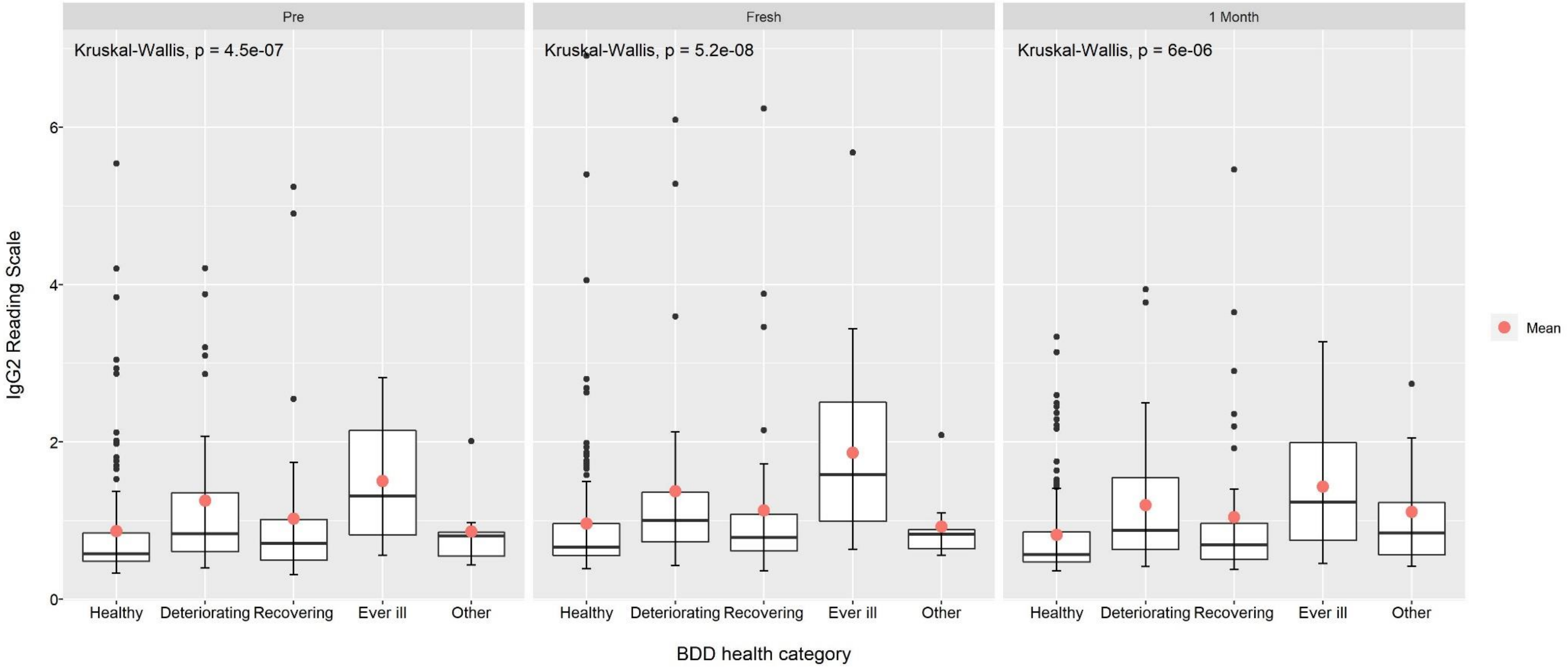

Figure 8. Indirect ELISA IgG2 reading according to BDD health category across the three data-collection time points

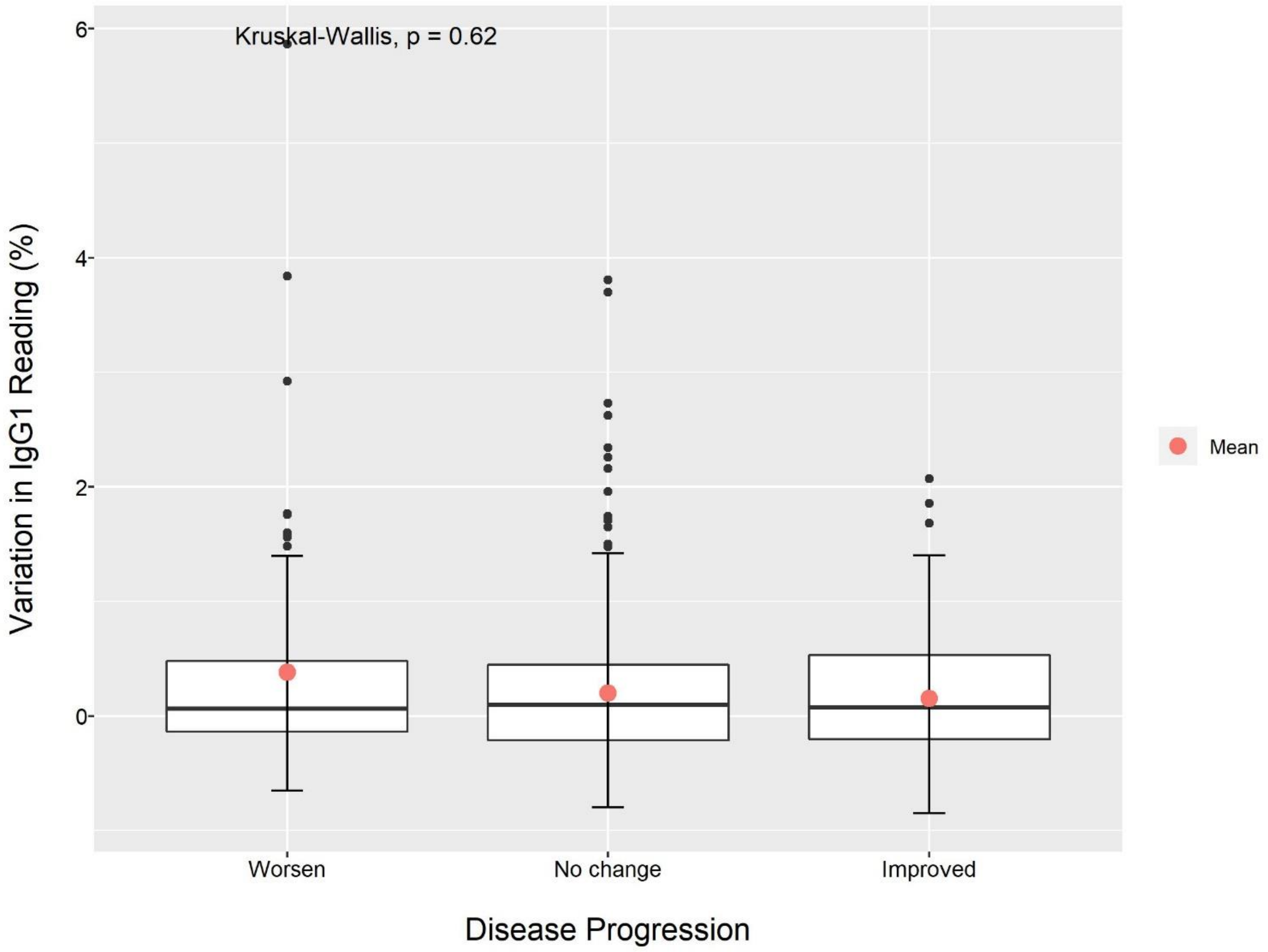

Figure 9. Variation in ELISA reading for IgG1 from time *t* to time *t-1* across the different disease progression categories

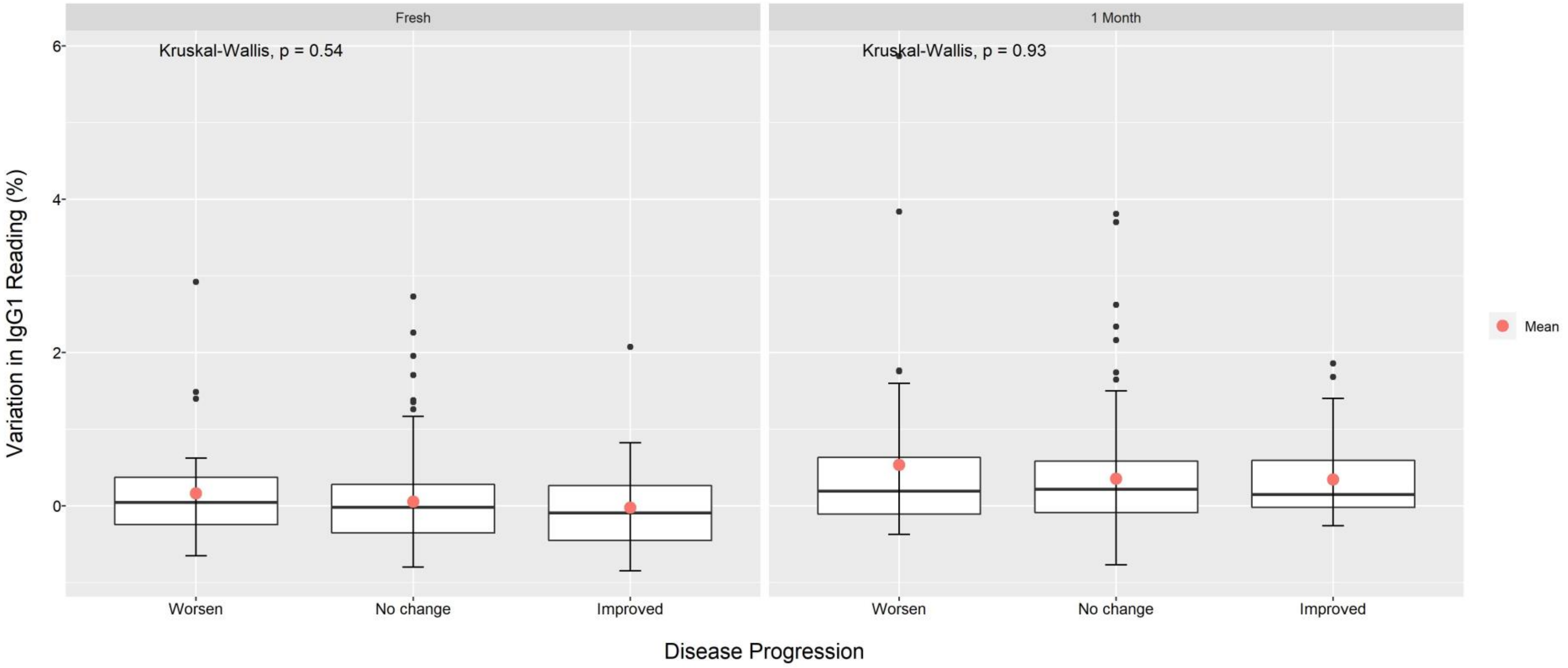

Figure 10. Variation in ELISA reading for IgG1 from time *t* to time *t-1* across the different disease progression categories, according to data collection time period

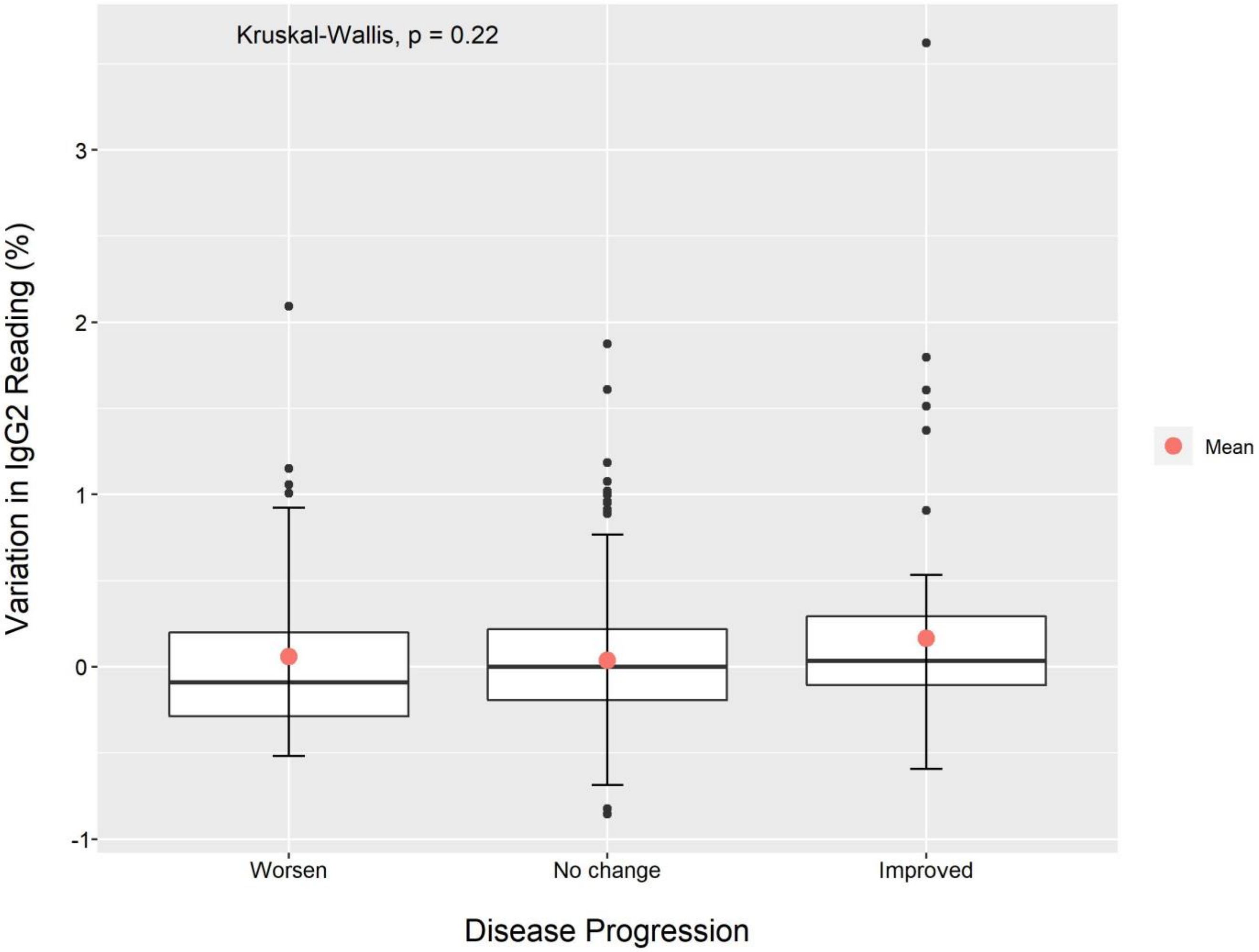

Figure 11. Variation in ELISA reading for IgG2 from time *t* to time *t-1* across the different disease progression categories

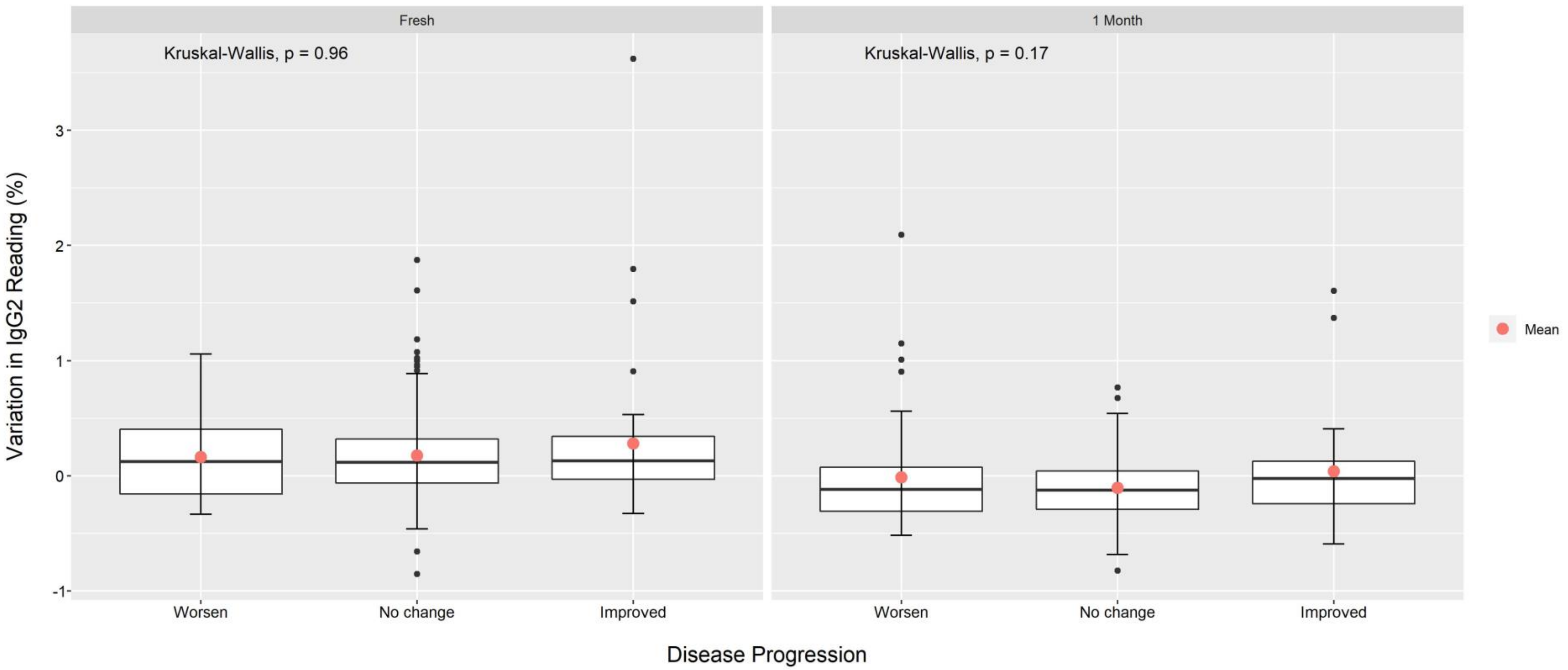

Figure 12. Variation in ELISA reading for IgG2 from time *t* to time *t-1* across the different disease progression categories, according to data collection time period
